# FOXM1 and Cancer: Faulty Cellular Signaling Derails Homeostasis

**DOI:** 10.3389/fonc.2020.626836

**Published:** 2021-02-15

**Authors:** Dhanya Kalathil, Samu John, Asha S. Nair

**Affiliations:** ^1^ Cancer Research Program-4, Rajiv Gandhi Centre for Biotechnology, Thiruvananthapuram, India; ^2^ Research Centre, University of Kerala, Thiruvananthapuram, India

**Keywords:** FOXM1, cell signaling, post-transcriptional regulation, post-translational regulation, miRNA

## Abstract

Forkhead box transcription factor, FOXM1 is implicated in several cellular processes such as proliferation, cell cycle progression, cell differentiation, DNA damage repair, tissue homeostasis, angiogenesis, apoptosis, and redox signaling. In addition to being a boon for the normal functioning of a cell, FOXM1 turns out to be a bane by manifesting in several disease scenarios including cancer. It has been given an oncogenic status based on several evidences indicating its role in tumor development and progression. FOXM1 is highly expressed in several cancers and has also been implicated in poor prognosis. A comprehensive understanding of various aspects of this molecule has revealed its role in angiogenesis, invasion, migration, self- renewal and drug resistance. In this review, we attempt to understand various mechanisms underlying FOXM1 gene and protein regulation in cancer including the different signaling pathways, post-transcriptional and post-translational modifications. Identifying crucial molecules associated with these processes can aid in the development of potential pharmacological approaches to curb FOXM1 mediated tumorigenesis.

## Introduction 

### A Conspectus on Forkhead Transcription Factors

The evolutionarily conserved, winged helix group of transcription factors are derivatives of bacterial helix-turn-helix motif. In eukaryotes, it was initially identified in the homeotic gene- forkhead, whose discovery led to the generation of a plethora of significant findings. Mutation of this gene caused defects in cephalic development of drosophila leading to a forkhead appearance and hence the name forkhead (1). Albeit winged helix transcription factors are conserved throughout evolution, during the course of its progression these groups of transcription factors have emerged with different binding specificities. Marsden et al. pointed out that the difference in binding specificities is the consequence of structural variations in the amino acid residues which lie in the immediate vicinity of the DNA binding motif (2). All forkhead transcription factors possess a characteristic 100 amino acid DNA binding domain which consists of three α-helices, three β-sheets and two large loops or wings that flank the third β-sheet (3).

Formerly, FOX proteins were designated as HFH, FREAC, and FKH. Subsequently, based on the phylogenetic analysis, these proteins have been divided into different subclasses (designated by a letter) and sub-subclass (denoted by an Arabic numeral). A unified nomenclature was introduced in 2000 which grouped the FOX proteins into different subclasses (FOXA-FOXS) based on sequence conservation. Human forkhead transcription factors are represented by uppercase letters whereas only the first letter is capitalized for mouse (4). FOXA, FOXC, FOXM, FOXP, FOXD, FOXE, FOXF, and FOXL have been shown to have a wide array of functions ranging from development to tumorigenesis (3, 5, 6).

### Forkhead Transcription Factor M1 (FOXM1)

**Figure 1:**
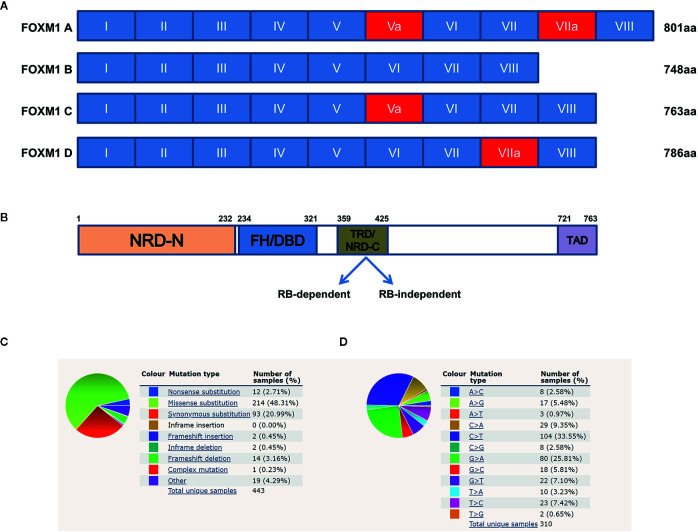
Structure of Forkhead transcription factor M1 (FOXM1) and its mutational overview. **(A)** Schematic representation of the human FOXM1 showing 10 exons (I–VIII), of which Va and VIIa (red) are alternatively spliced. **(B)** Domain structure of FOXM1C protein. TRD mediated repression of FOXM1 can be either Rb-dependent or independent. NRD-N, N-terminal repressor domain; FH/DBD, Forkhead box DNA Binding Domain; TRD/NRD-C, Trans-Repressor Domain/C-terminal Repressor Domain; TAD, Trans-Activation Domain. Numerical indicate amino acid positions. **(C, D)** COSMIC data (https://cancer.sanger.ac.uk/cosmic) showing summary of the types of mutation and frequency of substitution mutations for the base pair changes on the coding strand. Sample size used in the analysis from the database is 443 and 310 respectively.

FOXM1, previously named as HNF-3, HFH-11, MPP2, TGT3, INS1, PIG29, FKHL16, MPHOSPH2, LOC2305, or Trident is a member of the Forkhead Box (Fox) family of transcription factors (7). Human *FOXM1* gene consists of 10 exons which span approximately 25 kb on the 12p13.33 chromosomal band (7). *FOXM1* has four major splice variants namely *FOXM1A, B, C* and *D* which arise by differential splicing of exon Va and VIIa (**Figure 1A**). Among these, *FOXM1B* contain neither of the alternative exons whereas *FOXM1C* has retained the exon Va and *FOXM1D* has retained VIIa (8). *FOXM1B, FOXM1C*, and *FOXM1D* act as transcriptional activators, but *FOXM1A* which has retained both the exons has been reported to be the inactive variant, suggesting some dominant negative effect as it has retained the DNA binding capability (9). FOXM1 protein consists of N terminal repressor domain, forkhead box domain and C terminal transcriptional activation domain (**Figure 1B**). FOXM1 maintains cell homeostasis by controlling diverse biological processes such as proliferation, cell cycle progression, differentiation, DNA damage repair (DDR), tissue homeostasis, angiogenesis, apoptosis, redox signaling and drug resistance (10).

FOXM1 is involved in several pathophysiological conditions such as chronic obstructive pulmonary disease (COPD), asthma, acute lung injury (ALI), pulmonary fibrosis, pulmonary arterial hypertension (PAH) and cancer (11). This review mainly addresses the mechanisms by which FOXM1 is deregulated in cancer. A large amount of literature exists regarding FOXM1’s role in homeostasis and tumorigenesis, which the current review summarizes by primarily focusing on the altered upstream and downstream regulatory mechanisms in cancer. It is necessary to understand the various oncogenic pathways leading to the modulation of FOXM1 in response to environmental cues or oncogenic insults. This review sheds light on how integral and inherent FOXM1 is in the pathogenesis of cancer. As the review progresses the readers would obtain a clear view on multiple facets of FOXM1 in cancer and its impact on the homeostasis with special emphasis on the regulatory aspect of FOXM1 in cellular transformation.

### Genetic Alteration of FOXM1

FOXM1 is regarded as an oncogene due to its contribution in tumor initiation and progression whose expression has been shown to be elevated in various cancers (12) (13). Crucial mutations and gene copy amplification of FOXM1 have been observed at its loci 12p13.33 (https://cancer.sanger.ac.uk/cosmic) (14). Copy number alteration was observed in 29% of malignant peripheral nerve sheath tumors (MPNSTs) and also in breast cancers (15, 16). Barger et al. showed that mRNA and protein level alterations correlated with the copy number changes using the TCGA databases. Frequent amplification of FOXM1 was seen in various cancers among which testicular germ cell tumor had the maximum. Their analysis also revealed a correlation between aneuploidy and FOXM1 expression in TCGA pan-cancer aneuploidy clusters. Another study from the same group demonstrated that FOXM1 was found to be amplified in high-grade serous ovarian cancer (HGSOC) (17, 18).

COSMIC database has revealed several mutations and gene amplifications of FOXM1 across various cancers (**Figure 1C**). Synonymous mutation and missense substitution are observed to be the highest mutational events. Among the missense substitutions, C>T and G>A (**Figure 1D**) are found to be the most frequently occurring. These mutations have a wide range of FATHMM score (that predicts functional consequences of coding and non-coding variants). High FATHMM score (≥ 0.7) may predict a deleterious effect of these mutations. Most of these mutations may possibly alter the activity of FOXM1, but detailed studies need to be carried out to understand their effect at protein level, thereby the alterations in cellular physiology. The synonymous mutations do not have any deleterious effect on FOXM1 protein as this would not change the amino acid information. Nonsense mutations are observed in approximately 3% of the cases and these have a high pathogenic FATHMM score (>0.8). (https://cancer.sanger.ac.uk/cosmic) (14). Other genetic modifications of FOXM1 include gene fusion events like FOXM1/SLC2A14 (t (12;12)(p13;p13)), FOXM1/ANO2, FOXM1/DCP1B (19). Although SLC2A14 (hexose transporter), ANO2 (calcium activated chloride channel), DCP1B (core component of the mRNA decapping complex) have been shown to be associated with various cancers, their functional role as gene fusion products need to be studied in detail (20, 21). Apart from these genetic alterations, FOXM1 has been shown to be altered by various transcriptional and translational deregulations.

## Regulation of FOXM1

In order to maintain a homeostatic environment, FOXM1 expression and activity needs to be tightly regulated and coordinated by diverse signaling pathways. This involves the regulation by transcription factors (activation), repressors (repression) and the epigenetic modifications (activation or repression). Following transcription, cellular cues drive the processing of pre-mRNA molecules by machineries involved in post-transcriptional modification. Various studies have revealed that several miRNAs target FOXM1 and regulate its expression (22, 23). The diverse functions of FOXM1 have been further manifested through various post-translational modifications. Activation and repression of FOXM1 protein is mediated primarily by the phosphorylation status of respective amino acid residues. Apart from this, acetylation, SUMOylation and ubiquitinylation also contribute toward its activity and stability.

### Deregulated Cellular Signaling of FOXM1 in Tumorigenesis

**Figure 2:**
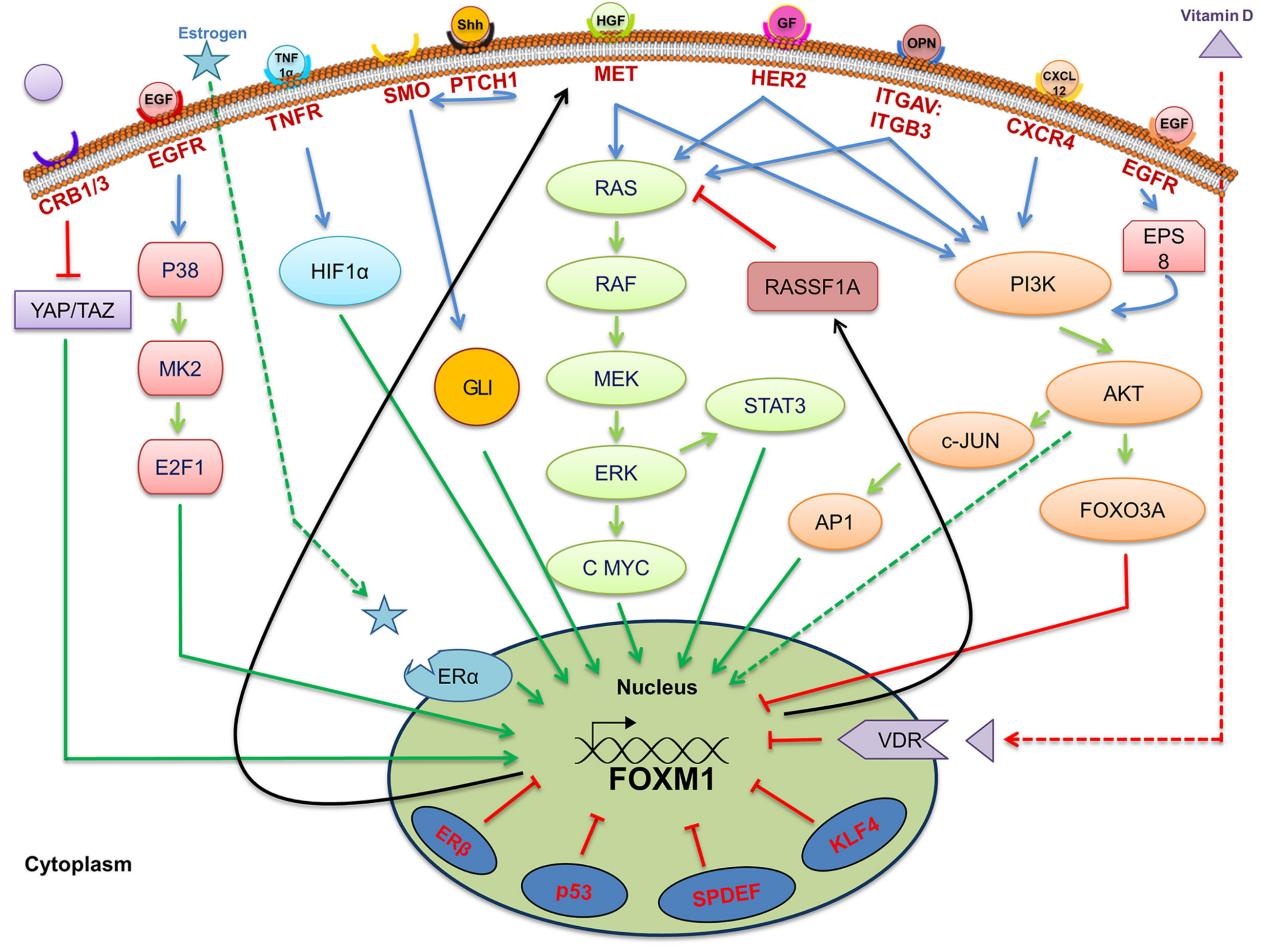
Signaling pathways associated with regulation of Forkhead transcription factor M1 (FOXM1). Pictorial representation of various upstream signaling factors that are involved in FOXM1 regulation. Ligands are indicated as colored objects above the bilipid membrane layer whereas receptors are labeled below the membrane. Cellular activators (green arrows), inhibitors (red arrows) and feedback loop (black arrows) indicate the mechanism of regulation of FOXM1.

FOXM1 is one of the major transcription factors altered in cancer by virtue of altered signal transduction pathways. Activation of FOXM1 *via* the altered KRAS signaling has been evident in the development of hepatocellular carcinomas and initiation of lung tumorigenesis in RAS driven tumors (24). Moreover existence of an inverse correlation between RASSF1A, a tumor suppressor of the RAS signaling pathway, and FOXM1 has been observed in the progression of colon cancer. Interestingly, a cross talk between FOXM1 transcription factor and RASSF1A (**Figure 2**) was also revealed from this study (25). RASSF1A, which is mainly altered *via* hypermethylation, protein degradation and point mutation has been widely observed in various cancers such as liver, breast, colon and bladder cancer (26–28). Another study has shown that FOXM1 is regulated by hepatocyte growth factor (HGF) *via* RAS/MEK/ERK, PI3K-AKT, and STAT molecules by the activation of mesenchymal epithelial transition (MET)-tyrosine kinase receptor. Interestingly, FOXM1 was observed to have direct binding site on MET promoter, thereby accelerating HGF/MET signaling. Presence of this positive FOXM1 and MET feedback loop accelerate pancreatic ductal adenocarcinoma (PDA) development (29). Whether FOXM1/MET overexpression have any association with metastasis of PDA need to be validated, however, its role in lymph node metastases of gastric cancer has already been established (30). Another signaling of FOXM1 *via* TGF-α/EGFR-STAT3-TESC was observed in cholangiocarcinoma progression (31).

One of the biomarkers for breast cancer detection is HER2, which has been shown to be altered in about 25% of the breast cancers. HER2 transmit the signals *via* RAS-MAPK or PI3K-AKT pathway, whose overexpression in breast cancer patients activates the expression of FOXM1 (32). It has been shown that FOXM1 nuclear positivity is well correlated with HER2 expression in breast cancer patients. This study also established an existence of a link between ER positive expression and FOXM1 in HER2 positive cases (33). Subsequent studies revealed FOXM1 overexpression was associated with aggressive phenotypes and poor overall and disease free survival in ER positive breast cancer patients (34). Moreover, FOXM1 expression in breast cancer cells has been shown to be controlled by a nuclear receptor, ERα which directly bind to the ERE like element on FOXM1 promoter. ERα, which is situated on nuclear membrane, could be activated by direct binding of estrogen or through a ligand independent mechanism. The latter mechanism involves key residues in the AF-1 domain of ER being activated by phosphorylation in response to signal transmission through RTKs, thereby promoting cell growth and survival (35). Further, HER2 and FOXM1 expression has also been observed to have correlation in colorectal cancer (36).

P38MAPK Pathway has been shown to be associated with drug resistance *via* FOXM1 expression. Epirubicin treated, MCF-7 breast cancer cells have shown induction of FOXM1 expression through P38MAPK–E2F1 axis. However, epirubicin resistant cells have shown constitutively high level of E2F1 and FOXM1 expression leading to the loss of drug sensitivity. It may be due to the fact that phosphorylation of E2F1 at serene 364 residue by P38-MK2 (mitogen-activated protein kinase (MAPK)-activated protein kinase 2; MAPKAPK2) axis led to the stabilization of E2F1 thereby regulating the target gene expression (37). Additionally, it was also shown that P38 could enhance FOXM1 expression independent of E2F1 by repressing JNK1. In contrast to these observations by De Olano et al., an earlier study by Millour J et al. has shown down regulation of FOXM1 expression at the onset of epirubicin treatment in MCF-7 cells by the modulation of E2F activity on the FOXM1 promoter by P53 (38). Additionally FOXM1 was shown to be associated with sorafenib drug resistance in liver cancer cells *via* AKT-CJUN-AP1- FOXM1 signaling (39).

FOXM1 expression in glioblastoma stem like cells has been shown to be elevated *via* FGFR signaling, wherein the expression of FGFR has been found to be elevated *via* α6-integrin and ZEB1/YAP1 transcription factor complex (40). A shorter overall survival was observed in glioblastoma multiforme (GBM) patients with the expression of α6-integrin, ZEB1/YAP1, FGFR1 and FOXM1 (41). Integrin αvβ3 receptor activation by osteopontin (OPN) has been found to upregulate FOXM1 expression in pancreatic cancer cells. OPN promotes the epithelial mesenchymal transition (EMT) and cancer stem cells (CSC) like properties of pancreatic cancer cells (PCCs) by activating the integrin αvβ3/Akt/Erk/FOXM1 cascade in a paracrine manner. The malignant phenotypes of PCCs could be induced by the OPN secreted from activated pancreatic stellate cells (PSC) in response to a hypoxic condition. Elevated OPN and FOXM1 expression reflects a poor clinical outcome in PCCs (42). Another study conducted in HEC1A cells also depicted the induction of FOXM1 expression by OPN (43).

A study by Chen et al. has shown that >60% of human breast cancer samples compared to adjacent normal breast tissues have high expression of epidermal growth factor receptor pathway substrate 8 (EPS8), enabling migration and motility (44). PI3K-AKT mediates, EPS8, dependent up regulation of FOXM1 which in turn leads to the activation of many of the cell cycle regulators, which includes CDC20, CDC25B, CDC25C phosphatases, Cyclin A, and Cyclin B among others. Additionally, FOXM1 regulates molecules associated with mitotic progression such as aurora-A kinase, polo-like kinase-1, centromere protein-A, E and F; stimulators of angiogenesis and motility including vascular endothelial growth factor (VEGF) and CXCL12. Chromatin immunoprecipitation assays in EPS8 overexpression background revealed an elevation in levels of acetylated histone H3 associated with the FOXM1 promoter (45). Recently EPS8 was shown as a novel interacting partner of FOXM1 which further established its role in enhanced cancer cell proliferation, migration and invasion (46). FOXM1 could also be activated *via* CXCL12 mediated PI3K/AKT-dependent mechanism in glioblastoma (47).

Sonic Hedgehog (Shh) Signaling activates FOXM1 expression through its effector molecule GLI1, a zinc finger transcription factor (48). Expression of FOXM1 correlates directly with the expression of patched-1 (PTCH1), smoothened (SMO) and GLI1 in various cancers (49, 50). Wang et al. have proved GLI1-FOXM1 interaction by depicting the direct binding of GLI1 to the FOXM1 promoter (51). Alteration in Hh signaling, thereby the expression of FOXM1 has been evident from reports on various cancers. It has also been observed that in some of the cancer cases even though GLI1 is altered at the initial stages, FOXM1 and GLI1 correlation is seen only when lymph node metastasis occurs. Hh and FOXM1 overexpression have been observed in cervical cancer tissue, wherein Shh, PTCH1 and GLI1 correlated with the pathological grade of the tumors and GLI1 and SMO correlated with the clinical stage of the tumors (50). Colorectal cancer cell proliferation was also enhanced in the background of Hh signaling and FOXM1 expression (51).

Salvador/Warts/Hippo (SWH) Pathway or in short Hippo signaling pathway regulates cell proliferation and apoptosis thus controlling the organ size in animals. Mechanical stress, G-protein-coupled receptor signaling, and oxidative stress are some of the upstream signaling for hippo pathway. This pathway also has been shown to be altered in cancer (52). The major protein kinase involved in the pathway is Hippo (Hpo) whose phosphorylation of downstream YAP is inactive during growth stimulation. Unphosphorylated YAP translocate to nucleus and interacts with TEAD to control gene transcription (53). In malignant mesothelioma, YAP/TEAD directly binds to the *FOXM1* promoter enhancing its gene expression (54). Moreover, FOXM1 upregulation has been observed in various soft tissue sarcoma subtypes which led to an increased cell proliferation (55). ROCK/YAP/FOXM1 axis has been observed in airway smooth muscle cell proliferation, migration, and contraction which is induced by S1P binding to S1PR_2/3_ (56). YAP/TEAD-FOXM1 signaling axis has also been associated with the expression of chromosomal instability signature genes CIN25 and CIN70 expression in hepatocellular carcinoma (HCC) (57). Studies in breast cancer cells MDAMB 231 have also shown FOXM1 to modulate proliferation, clonal expansion, migration and stemness in YAP1 dependent manner (58). Through an integrated transcriptomic, proteomic, and drug screening approach, YAP-FOXM1 axis was identified as the driver of EMT-associated EGFR-TKI (Tyrosine Kinase Inhibitor) resistance by increased abundance of spindle assembly checkpoint (SAC) proteins, including polo-like kinase 1 (PLK1), aurora kinases, survivin, and kinesin spindle protein (KSP).

FOXM1 is also regulated by another stress signaling pathway, TNFα/ROS/HIF-1 in hepatocellular carcinoma. The existence of a positive correlation between HIF1α and FOXM1 in HCC patients reflects poor prognosis, aggressive tumors, and high recurrence rate (59). An increased incidence rate of cancer in colon, breast, prostate and ovarian has been inversely correlated with the vitamin D deficiency (60). An inverse correlation between vitamin D receptor expression and FOXM1 in pancreatic ductal carcinoma patients was observed by Li Z et al. Pharmacological activation of Vitamin D receptor (VDR) by vitamin D and its analog has led to the suppression of FOXM1 signaling as evidenced from the expression of its downstream targets such as cyclin D1, CMYC, SKP2, CD44 and c-MET (61). Overall representation of the upstream signaling of FOXM1 has been given in **Figure 2**.

### Post-Transcriptional Alterations of FOXM1 in Cancer

The fate of mRNAs is decided by various intricate mechanisms which include capping, splicing, polyadenylation and rate of nuclear export. All these processes are controlled by various protein complexes and enzymes to maintain homeostasis.

#### Deregulation in Splicing Machinery

**Figure 3:**
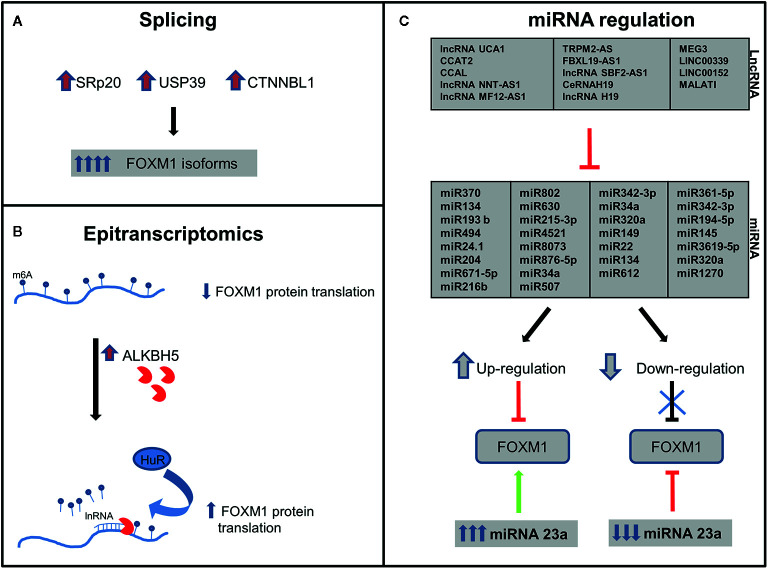
Post-transcriptional regulation of Forkhead transcription factor M1 (FOXM1). FOXM1 regulation by **(A)** Splicing-elevation of splicing factors lead to upregulation of FOXM1 isoforms **(B)** Epitranscriptomics-upregulation of ALKBH5 leads to increased FOXM1 translation and **(C)** lnRNA/miRNA-FOXM1 gene expression is regulated by miRNA which in turn are regulated by lnRNA (details of specificity are described in **Table 1**).

Pre-mRNA molecules undergo splicing to generate various isoforms of a particular gene. Approximately 70% of the human genes undergo splicing events and generate diversity in the protein profile. SRp20 (SFR S3), a splicing factor that regulates FOXM1, PLK1, and Cdc25 B transcripts, has been observed to be overexpressed in ovarian, cervical, AML, lung, breast, stomach, skin, bladder, colon, liver, thyroid, and kidney cancers (62, 63). Precise regulation by splicing is quintessential to maintain cellular homeostasis by FOXM1 as evidenced from the G2/M phase arrest due to SRp20 downregulation. Accumulation FOXM1A and downregulation of both FOXM1B and FOXM1C have been observed upon knockdown of SRp20 (63). In addition, USP39, a component of the spliceosome, also regulates FOXM1 pre-mRNA processing. High expression of USP39 has been observed in human HCC and FOXM1 is found to be one of the molecules overexpressed (64). Inhibition of USP39 by siRNA has downregulated FOXM1 and in turn led to tumor volume reduction in xenograft model of HCC (65). Another protein involved in FOXM1 splicing is a nuclear protein, CTNNBL1 which has been found to be associated with the Prp19 complex of the spliceosome. This protein is involved in regulating the splicing events of FOXM1 in ovary and its elevated expression was reported in ovarian cancer. FOXM1B and C protein levels were high in CTNNBL1 overexpressed cells, whereas a decreased expression has been observed in knockdown ovarian cancer cells (66). These splicing mechanisms have been illustrated in **Figure 3A**.

#### Deregulation in Epi-Transcriptomics

Incorporation of N6-Methyladenosine (m6A) into the pre-mRNA molecules by m6A methyltransferases-METTL3 is one of the most important epi-transcriptomic modifications present in eukaryotes that play important role in various physiological processes and disease states. This modification could be removed either by FTO (fat-mass and obesity-associated protein) or ALKBH5 (α-ketoglutarate-dependent dioxygenase alkB homolog 5) (67). ALKBH5 expression has been reported to be high in breast cancer, GBM, ovarian cancer, pancreatic cancer and gastric cancer (68). A study by Zhang et al. had demonstrated FOXM1 as a target of ALKBH5 and also identified a long non-coding RNA antisense to FOXM1. Interaction of long noncoding RNA with FOXM1 promotes the recruitment of ALKBH5 to the FOXM1 nascent transcripts. This in turn leads to the binding of HUR, an RNA binding protein thereby resulting in protein translation (**Figure 3B**). Blockade of these molecules by shRNA interrupted the proliferation of GSCs and GBM (69). So it would be speculated that the deregulation on these molecules in cancer may affect the expression of FOXM1 transcription factor.

#### Deregulation of FOXM1 by miRNA and lncRNA

Approximately 60% of genes encoded in the human genome are regulated by miRNAs (70). It has been shown that many physiological processes such as development, apoptosis, EMT, proliferation and stem cell maintenance are tightly regulated by miRNAs (71). Studies related to FOXM1 and miRNA so far has generated ample knowledge regarding potential miRNAs which inhibit FOXM1 post-transcriptionally.

The miRNA which target and regulate FOXM1 expression has widely been altered in various cancers. These miRNAs either function as tumor suppressors or oncogenes. FOXM1 is not only altered through the direct binding of miRNA to the 3’UTR but also by indirect effect of miRNAs on upstream regulatory molecules of FOXM1. Upregulation of FOXM1 by inhibiting its miRNA reflected in phenotypes such as proliferation, invasion and migration (**Table 1**). CagA, a virulence factor of *Helicobactor pylori* promotes the expression of FOXM1 by downregulating miRNA 370 in gastric cancer (73). Another mechanism of FOXM1 miRNA downregulation is by the hypermethylation of its own promoter (78).

**Table 1:** List of miRNAs that regulate Forkhead transcription factor M1 (FOXM1).

Sl. No.	miRNA	Loci	D/U	Associated Cancer	Highlights
1.	miR370	14q32	**↓**	Osteosarcoma (72), Gastritis and Gastric cancer (73), Acute myeloid leukemia (22)	Regulates cell proliferation, invasion and migration (72). Downregulation in 77% (37/48) of patient samples (AML) (73). Epigenetic silencing of miR370 in leukemic cells (22).
2.	miR134	14q32.31	**↓**	Esophageal Squamous cell carcinoma (ESCC) (74), Lung adenocarcinoma (75), Non-small-cell lung carcinoma (NSCLC) (76) Hepatocellular carcinoma (HCC)	Could inhibit the migration and invasion of ESCC cells (74). Regulates proliferation and metastasis *via* **LncRNA MFI2-AS1**/miR134/*FOXM1*. **LncRNA MFI2** overexpression associated with poor prognosis and advanced stage among patients (77).
3.	miR193b	16p13.12	**↓**	Prostate cancer	Hypermethylation of the promoter has been observed in cancer. Inhibits migration and invasion in prostate cancer cell lines (78).
4.	miR494	14q32.1	**↓**	Pancreatic cancer	PDAC metastasis and reduced survival times of patients correlated with reduced expression of miR 494. Could efficiently inhibit cell migration and invasion in pancreatic cells (79).
5.	miR24-1	9q22.32	**↓**	Bladder cancer	Induction of miR24-1 resulted in inhibition of cell proliferation, cell cycle arrest and increased apoptosis (80).
6.	miR204	r9q21.12	**↓**	Esophageal cancer (EC)	Inverse correlation with EMT phenotype of EC cells (81). Functions as tumor suppressor or oncogenes (82).
7.	miR671-5p	7q36.1.	**↓**	Breast cancer cells	Expression is inversely proportional to the invasive and metastatic properties of the cancer cell (83). Gradual reduction of mRNA in progression of normal epithelial to atypical ductal hyperplasia (ADH), to ductal carcinoma *in situ* (DCIS), and then invasive ductal carcinoma (IDC) (84).
8.	miR216b	2p16.1	**↓**	Hepatocellular carcinoma Cervical cancer (85)	Administration to HepG2 cells has produced a prominent cell cycle arrest phenotype and apoptosis (86).
9.	miR802	21q22.12	**↓**	Breast tissues	Proliferation of MCF7 breast cancer cells (87).
10.	miR23a	19q13.10	**↑**	Breast cancer	Function as tumor suppressor or Oncogenes. Potential putative biomarker of breast cancer (88).
11.	miR630	15q24.1	**↓↑**	Hepatocellular and bladder cancer (89–91) (Overexpression) Gastric cancer (downregulation)	Function as oncogene or tumor suppressor gene
12.	miR215-3p	1q41	**↓**	Colorectal cancer	Functions as oncogene as well as tumor suppressor gene. Inversely proportional to lymph node metastasis in colon cancer (92).
13.	miR4521	17p	**↓**	Medulloblastoma	Accelerates proliferation and invasion of several medulloblastoma cell lines (93).
14.	miR8073	13q34	**↓**	Colon, breast, and pancreatic cancer	Increased tumor growth (94).
15.	miR876-5p	9p21.1	**↓**	Glioblastoma (GBM) Breast cancer	Accelerated cell proliferation, reduced apoptosis and increased migration and invasion capabilities of GBM cells (95). Regulates cell proliferation and cell apoptosis *via* **FBXL19-AS1/**miR876-5p/*FOXM1* axis (96).
16.	miR34a	1p36.22	**↓**	Esophageal Squamous cell carcinoma (ESCC), Liver cancer (LC) Hepatocellular carcinoma (HCC)	Accelerates cell proliferation and cell migration (97). DNMT1 causes miR34a promoter methylation and suppression, leading to FOXM1 upregulation and promotes LC stemness (98). Regulates cell differentiation, and metastasis *via* **CCAT2**/miR-34a/*FOXM1*. FOXM1 activates CCAT2 transcription (99).
17.	miR507	Xq27.3	**↓**	Melanoma cells	Regulates FOXM1 expression *via* **lnRNA UCA 1/**miR 507/*FOXM1* axis (100).
18.	miR342-3p	14q32.2	**↓**	Cervical Cancer GBC tissues Gallbladder cancer	Control cell proliferation, migration and invasion in cervical cells by regulating the expression of FOXM1 (101). **lnRNA H19** and miR-342-3p expression are inversely proportional (102). **CeRNA H19** expression regulates cell proliferation and invasion *via* H19/miR342-3P/*FOXM1* axis (102).
19.	miR320a	8p21.3 8p21.3	**↓**	Renal Cell Carcinoma (RCC) Human umbilical vein endothelial cells (HUVECs)	RCC cell proliferation, invasion and migration (103). Regulates HUVEC proliferation *via* **MALAT1**/miR-320a/*FOXM1* (104).
20.	miR149	2q37.3	**↓**	Gastric cancer	Regulates proliferation and metastasis *via* **CCAL/**miR-149/*FOXM1*axis (105).
21.	miR22	17p13.3	**↓**	Lung Squamous cell carcinoma	Regulates proliferation, invasion, migration and metastasis *via* **LnRNA NNT-AS1**/miR 22 (106).
22.	miR612	11q13.1	**↓**	Gastric Cancer	Regulates proliferation, migration, invasion and radioresistance *via* **TRPM2-AS**/miR 612/*FOXM1* axis (107).
23.	miR361-5p	Xq21.2	**↓**	Cervical Cancer Osteosarcoma cells	Regulates cell proliferation *via* **lncRNA SBF2-AS1**/miR361-5p/*FOXM1*. Elevated expression of lncRNA SBF2-AS1 was associated with advanced FIGO stage and lymph node metastasis of CC patients (108). Regulates proliferation and migration *via* **MEG3**/miR-361-5p/*FOXM1* axis (109).
24.	miR194-5p	1q41	**↓**	Gastric cancer, Colorectal adenocarcinoma	Regulates proliferation invasion, and migration *via* **lncRNA H19**/miR-194-5p/*FOXM1* axis (110).
25.	miR145	5q32	**↓**	Non-small-cell lung carcinoma (NSCLC)	Regulates proliferation, invasion and apoptosis *via* **LINC00339**/miR145/*FOXM1* axis (111).
26.	miR3619-5p	22q13.31	**↓**	Thyroid cancer	Regulates proliferation and apoptosis through **LINC01410**/miR-3619-5p/*FOXM1* (112)
27.	miR1270	19p12	**↓**	Rheumatoid arthritis (RA)	Regulates proliferation and apoptosis *via* **LINC00152**/miR-1270/*FOXM1* (113).

Downregulation (D) or Upregulation (U) (indicated by arrows) of miRNAs alters FOXM1 expression in various cancers. List also shows the outcomes of altered miRNA expression and lncRNAs (in bold) that regulate miRNAs.

Recent studies on FOXM1 miRNAs have revealed several prospective targets for cancer treatment. It has been shown that miRNA inhibitors have the ability to re-sensitize drug resistance cells to chemotherapy. This could have positive implications as FOXM1 is a major molecule in drug resistance mechanism. The drug resistance phenotype in cancer cells has been observed to be reversed by the overexpression of miRNA 134 (75), whereas miRNA 320 enhanced radiosensitivity by directly targeting FOXM1. Majority of the reported miRNAs has a potential to be considered as a prognostic and diagnostic marker. **Table 1** shows an overview of the reported miRNAs of FOXM1. Despite several miRNA studies revealing its association with cancer, only few have reported the regulatory mechanisms of miRNA. A study indicated miR 135a has been found to be transcribed by FOXM1 transcription factor and metastasis suppressor1 (MTSS1) (114). It also transcriptionally enhanced the expression of miR-1306–3p, a plausible biomarker for clinical prognosis of HCC.

Long noncoding RNA (lncRNA) are transcripts with lengths exceeding 200 nucleotides. They are critical in tumorigenesis, chromatin remodeling, and post-transcriptional regulation. Last few years have shown growing evidence of lncRNA-miRNA-mRNA axis in regulation of several proteins. miRNA 507 and lncRNA UCA1 cooperatively regulate FOXM1 expression in melanoma cells. UCA1 has a direct binding site on miRNA 507 and has led to the existence of a negative correlation between lncRNA and miRNA 507 (100). FRLnc1, a long non coding RNA has been shown to be upregulated in 49% (20/41) of gastric cancer cases by positively regulating the expression of FOXM1 leading to enhanced cell migration. Apart from these, many lncRNA like H19, LINC00339, LINC01410, Metastasis-associated lung adenocarcinoma transcript 1 (MALAT1), SBF2-AS1, FBXL19-AS1, MEG3, TRPM2-AS, MFI2-AS1, NNT-AS1, CCAL, modulate the expression of FOXM1 *via* miR-342-3p and miR-194, miR-145, miR-3619, miR-320a, miR-361-5p, miR-876-5p, miR-612, miR-194-5p, miR-134, miR-22, and miR-149 respectively in various cancers. Few studies also demonstrated a positive feedback loop between FOXM1 and LncRNA like LINC01410, CCAT2, PVT1 and TUG1 (112, 115, 116) (**Table 1** and **Figure 3C**).

### Post-Translational Modification of FOXM1 in Cancer

**Figure 4:**
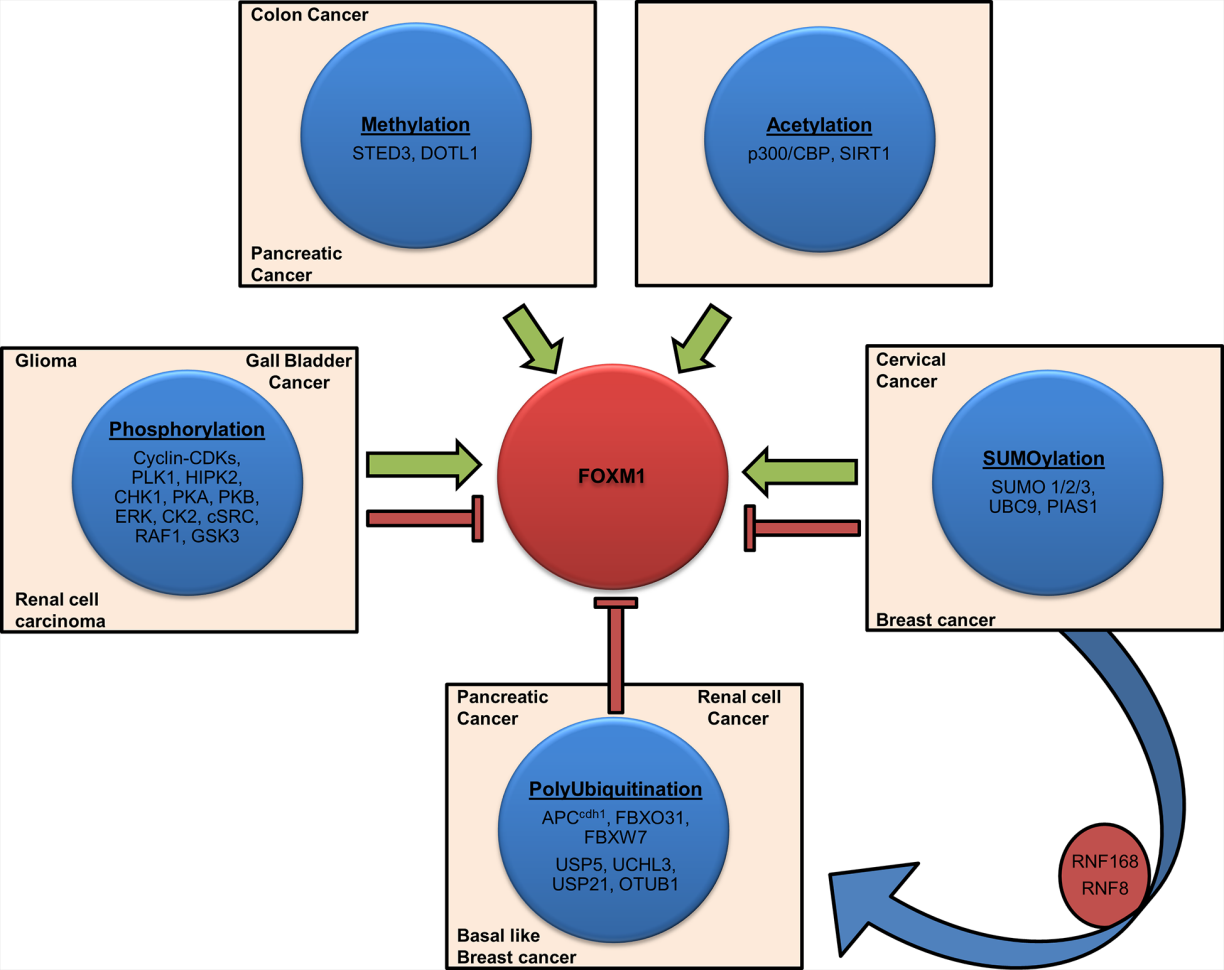
Post-translational modifications of Forkhead transcription factor M1 (FOXM1). Schematic representation shows various factors involved in FOXM1 post-translational modifications (PTMs). Activation (Methylation, Acetylation), inhibition (Polyubiquitination), contextual (Phosphorylation, SUMOlyation) and crosstalk are depicted by representative arrows. Cancers associated with the altered expression of these factors have also been indicated.

Post-translational modification (PTM) of FOXM1 includes methylation, phosphorylation, acetylation, SUMOylation and ubiquitination. Appropriate activation, inactivation and degradation of this protein are vital for the execution of various cellular processes and maintenance of homeostasis. As FOXM1 plays a major role in many cellular processes, any deregulation in its activity by way of faulty PTMs may facilitate the process of tumorigenesis. Overall view of the FOXM1 PTMs and the factors associated are depicted in **Figure 4**.

#### Methylation 

Methylation is a PTM in which methyl groups are added to the lysine and arginine residues of proteins. Methyltransferase-SETD3 modify FOXMI under normoxic conditions and hamper its transcriptional activity (117). A proteomic study had revealed K278 and K282 as the plausible sites of methylation on FOXM1 (118). On the other hand, increased transcriptional activity of FOXM1 was mediated through the dimethylation of H3K27 by DOT1L in pancreatic and colon cancer. The tumor promoting effect in these cancers was mediated *via* WNT5A, a downstream target of FOXM1 (119).

#### Phosphorylation

**Figure 5:**
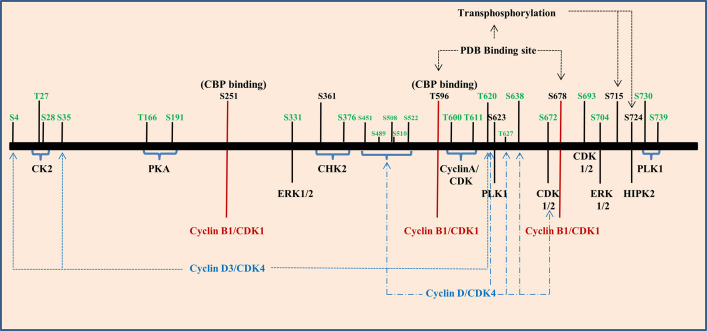
Phosphorylation of Forkhead transcription factor M1 (FOXM1) is regulated by several kinases. Graphical representation of various kinases that regulate the different phosphorylation sites of FOXM1B (black) and C (green).

Phosphorylation is a process in which phosphate group is covalently attached to the respective amino acid of a protein substrate by an enzymatic reaction catalyzed by various protein kinases. Major protein kinases involved in this process are serine threonine protein kinase, tyrosine protein kinase, protein kinase A, B, and C. FOXM1 phosphorylations are mostly carried out by ERK, Cyclin dependent kinases (CDK), Polo like Kinase, and protein kinase A, B, and C (**Figure 5**). Transcriptional activity of FOXM1 mostly depends on the activation by Ras-mitogen-activated protein kinase (MAPK) signaling pathway, which activates FOXM1 through Cyclin-CDKs (120). Phosphorylation of FOXM1 at various residues happens to be in a cell cycle dependent manner (121). It has been shown that FOXM1B phosphorylation starts at the G_1_ phase (4 h post serum addition) and progresses through the S phase (12 h) and G2 phase (18 to 22 h) of the cell cycle which directly correlated with the transcriptional activity of this protein (122). Another study also has reported FOXM1 activity to be low in cells synchronized at the G_1_/S transition and increased only as the cells entered the G_2_ phase after release from the G_1_/S block (10 to 12 h after release), whereas expression of FOXM1 was relatively constant. Induction of FOXM1 activity in G_2_ was not due to enhanced DNA binding, but due to differences in its phosphorylation status as FOXM1 was already bound to its target promoters in G_1_/S phase of the cell cycle (123).

CyclinD1/D3 and CDK4/6 complexes play a role in phosphorylating FOXM1 at initial stages of cell cycle and they have also been found to be associated with neoplastic growth. These phosphorylation events aid to suppress the senescence program hence they may accelerate tumorigenesis (124). When cells enter S and G2 phase, Cyclin E/A and CDK2 complex phosphorylates FOXM1C at Thr600, Thr611 and Ser638 and regulate the transcriptional activity (125). Three potential CDK 1/2 sites were observed in the FOXM1B protein which includes amino acid residues at 585, 596 and 657. Among these T596 residue has been recognized as a critical CDK1 phosphorylation site within the activation domain of FOXM1B. CDK-dependent phosphorylation stimulates the FOXM1B transcriptional activity, which has correlated with binding to the CREB-binding protein (CBP), the transcriptional co-activator. Mutation of T596 abolishes binding to CBP and inhibits transcriptional activity of FOXM1B (122). Mutation of the other two sites showed only a marginal decrease in the transcriptional activity of FOXM1B. Cyclin-binding motif (LXL motif) located at residues 639 to 641 has been responsible for the binding of CDK- Cyclin and both CDK1 and CDK2 were shown to be associated with FOXM1B. Mutation of the Cyclin-binding motif (Leu-641-Ala) has resulted in the elimination of both CDK1 and CDK2 binding. Phosphorylation of S251 in the forkhead box domain of FOXM1B stands as a critical event for activation of its transcriptional activity at the G_2_/M phase by CDK1. Chen et al. also proved that S251 residue may be required for the nuclear localization of FOXM1 and its DNA binding. Disruption of these sites inhibits phosphorylation of FOXM1 and reduced transcriptional activity (126).

Entry of FOXM1 in to G2/M triggers polo-like kinase 1(PLK1) mediated phosphorylation. PLK1 Binding domain (PBD) recognizes S-pS/pT-P/X22,24 consensus sequence. By transferring phosphate group from ATP to substrates, PLK1 control various G2/M associated cellular processes, namely centrosome maturation, checkpoint recovery, spindle assembly, cytokinesis, and apoptosis. Also, PLK1 phosphorylation sites in FOXM1B serves as a regulator for its repressor function in G1 phase and activator function in S and G2/M phase (127). FOXM1 has two potential PBD-binding sites (T596 and S678) at its C terminal region and phosphorylate S715 and S724 present within the TAD region of FOXM1 (128). It has been shown that PLK1 and FOXM1 expression were positively correlated in renal cell carcinoma cell lines. This study has also shown that the down regulation of PLK1 by siRNA resulted in reduced expression of FOXM1 (129). PLK1 mediated phosphorylation further promotes phosphorylation by MELK leading to increased expression of mitotic genes in glioma stem cells (130). It has been shown that shorter overall survival of gall bladder cancer patients (GC) were significantly associated with FOXM1, PLK1, and NEK7 (131). Moreover, esophageal adenocarcinoma cell lines and tissues showed increased expression of FOXM1 and PLK1 than the normal counterpart and barrettes metaplasia. In addition they have also observed increased expression of FOXM1A and FOXM1B in most of the samples but heterogeneity was observed in FOXM1C’s expression (132). The histological grading of renal cell carcinoma has been correlated with the expression of FOXM1 and HIPK2 wherein HIPK2 phosphorylates FOXM1 at S724 (133). Two ERK phosphorylation sites have been identified in FOXM1C at residues 331 and 704 respectively and its phosphorylation *via* MAPK signaling accelerate nuclear translocation of FOXM1C and bring its G2/M regulatory effect (120).

The role of FOXM1 in DNA damage response has been evident from various studies. At the onset of DNA damage, phosphorylation of FOXM1 by CHK2 kinase ensures a proper DNA damage response through the stabilization of FOXM1. In FOXM1B, S361 residue marks the phosphorylation site for CHK2 and has been established to be conserved between mouse and humans (134). Apart from the role of Cyclin–CDK, CHK2 and PLK1, activation of FOXM1C was found to have occurred by the action of protein kinase CK2, cAMP dependent protein Kinase A, c-SRC and RAF-1. PKA phosphorylate substrates by recognizing R/K-R/K-X-S/T consensus sequence. FOXM1C has two such sequences around T160 and S190 at the N terminal region. Likewise, protein kinase CK2 recognizes the motif S**/**T-X-X-E/D. Phosphorylation of FOXM1C by CK2 at residues T27 and S28 results in its activation by removing the inhibitory phosphorylation. c-SRC and RAF1 are two other kinases also found to be involved in the activation of FOXM1C through N terminal region (135). **Figure 5** summarizes various phosphorylation sites on FOXM1B and C that has been discussed.

#### Acetylation

Acetylation of histones or transcription factors represents a transcriptionally active state in most cases. This process involves the transfer of acetyl group from donors to the α-amino group of the first amino acid residue of a protein or to the ϵ-amino group of a lysine residue by lysine acetyl transferases. Apart from the role in the transcriptional activation, acetylation has also been associated with subcellular localization and stability of the protein. Acetylation of FOXM1 by p300/CBP at lysines K63, K422, K440, K603 and K614 has been noted to be essential for its transactivation of the target genes. This modification of FOXM1 increases during the S phase and remains elevated throughout the G2 and M phases (136). Apart from its increased transcriptional activity, acetylation of FOXM1 resulted in stabilization of the protein. Acetylation of FOXM1 follows a peak curve; levels increased initially in the S phase (4 h), elevated in the early G2 phase (8-10 h), reached maximum levels at the late G2/M phases (12-14 h), and then drops down when entering G1 phase (16-22 h). Observation of Lv et al. indicated that although the interaction between FOXM1 and p300 was initiated at the S phase, it reached the peak at G2/M phase. Notably, optimal binding of FOXM1 and CDK1 depends upon the acetylation of FOXM1 which further contributes toward its activation by phosphorylation. FOXM1 Thr 596 CDK phosphorylation site can recruit p300/CBP to the FOXM1B transcriptional activation domain. This interaction leads to acetylation of FOXM1 and thereby it’s increased transcriptional activity. Moreover, FOXM1-dependent transcription is negatively affected by deacetylases like SIRT1. Tumor growth potential was significantly reduced in acetylation deficient mutants compared to wildtype FOXM1 (136).

#### SUMOylation

SUMOylation is a highly dynamic and reversible post translational modification that modulates FOXM1 activity in a context dependent manner. It involves covalent attachment of small ubiquitin-related modifier (SUMO) to specific lysine residues and thus regulating various aspects such as its subcellular localization, cell cycle progression, transcription, and DNA repair events (137). ψKXE consensus motifs for SUMOylation are present throughout FOXM1 at amino acid positions 201, 218, 341, 445, 463, and 480. SUMOylation of ψKXE motifs on the transactivation domain of FOXM1C promoted cytoplasmic accumulation and degradation by APC^Cdh^ ubiquitin ligase in breast cancer cells. It was also reported that epirubicin resistant cells are refractory to the SUMOylation, hence its transcriptional activation rather than destabilization (138). SUMO1 mediated inactivation of FOXM1 was shown to be reverted by PLK1 activity (139).

A study by Jaiswal et al. reported that SUMO conjugating enzymes, UBC9 and PIAS1 accelerates destabilization and nucleo-cytoplasmic shuttling of FOXM1. They also reported that HPV16 E7 oncoprotein can prevent SUMOylation of FOXM1B by impairing its interaction with UBC9 leading to its increased transcriptional activity in HPV positive cervical cancer cell lines (140). In contrast to the above mentioned studies, Wang et al. showed an increased activity of FOXM1 by SUMO1 mediated SUMOylation in breast cancer cells (141). In addition, Schimmel et al. demonstrated transcriptional activation of FOXM1 by SUMO2 in wild type compared to the SUMOylation deficient FOXM1 mutant. Their results indicated that SUMOylation blocks the dimerization of FOXM1, thereby relieving FOXM1 auto repression (142).

#### Ubiquitination

Orderly synthesis and degradation of proteins are necessary for the proper functioning of the cell. Degradation of the proteins is initiated by the addition of ubiquitin molecules (polyubiquitination) to the lysine residue by ubiquitin activating and conjugating enzymes (E1 and E2), and their recognition by E3 ubiquitin ligases (143). Studies on APC^Cdh1^, an E3 ubiquitin ligase, showed its involvement in degrading FOXM1 in late M and early G1 phase of cell cycle. This degradation is brought about by D box and KEN box APC^Cdh1^ recognition motifs present at the N terminal region of FOXM1 (144). Role of other ubiquitin ligases like FBXO31’s in ubiquitinating and degrading FOXM1 have also been demonstrated in G2/M transition (145). Additionally, the role of deubiquitinase enzymes like USP5, UCHL3 and USP21 have been demonstrated to stabilize FOXM1 in pancreatic cancer, and basal like breast cancer (BLBC) respectively (146–148). Deubiquitination of FOXM1 by UCHL3 was also shown to promote pancreatic cancer progression and gemcitabine resistance (146). Also, a recent study showed that OTUB1 mediated deubiquitination of FOXM1 promotes renal cell carcinoma *via* upregulation of ECT 2 (149).

Protein quality control (PQC) mostly relies on an efficient spatiotemporal regulation of proteins by crosstalk between various cellular machineries. Kongsema et al. showed that SUMOylation was a prerequisite to recruit RNF168, an E3 ubiquitin ligase, to degrade FOXM1 in response to genotoxic stress in MCF-7 breast cancer cells. Further, their study also revealed that RNF168 works in cooperation with RNF8 as it only has a conventional ubiquitin-interacting motif and not a SUMO interacting motif (150). Chen et al. also showed that FOXM1 degradation was induced by FBXW7 dependent ubiquitination upon S474 phosphorylation by the GSK3-axin complex. However this process was inhibited by the Wnt signaling activation and subsequent interaction with USP5 thereby leading to the stabilization of FOXM1 in glioblastoma multiforme (151).

## Regulation by FOXM1

FOXM1 transcriptionally regulates the expression of a plethora of genes involved in various cellular processes such as cell cycle, DDR, senescence, apoptosis, migration, invasion, oxidative stress, and drug resistance. This protein has been found to be altered in various cancers by contributing to all the hallmarks of cancer. Thus it can be considered to be a potential target for precision cancer treatment modalities.

**Figure 6:**
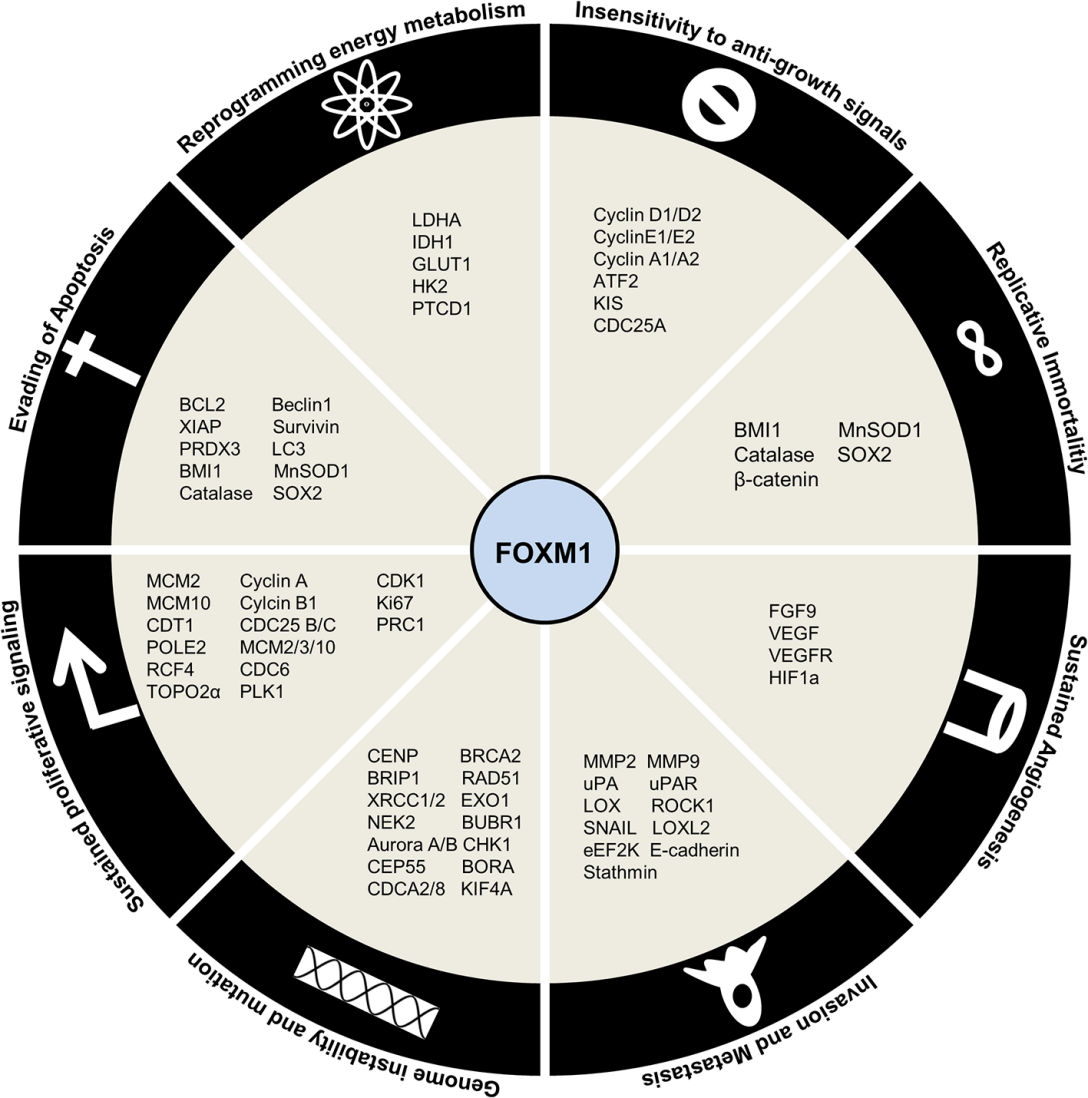
Regulation by Forkhead transcription factor M1 (FOXM1) in cancer. Schematic representation of downstream targets of FOXM1 that contribute to Hallmarks of Cancer.

FOXM1 maintains sustained proliferation by modulating the expression of crucial cell cycle proteins. FOXM1 was shown to mediate G1 and S phase transitions by activation of CyclinD1 (152), Cyclin E2 (124), Cyclin A2 (153), ATF2 (154), KIS (155), CDC25A (156) and reduction of CDK inhibitors p16, p27^Kip1^, p21^Cip1^ (insensitivity to anti-growth signals) through its degradation by SKP2 and CSK1 (SCF ubiquitin ligase complexes) (**Figure 6**) (157). It also regulates DNA replication initiation by MCM2, MCM3, MCM10, CDT1 (124), CDC6 (158) and progression by POLE2, RFC4 (159), TOP2A (160), whose deregulation could lead to loss of fidelity. G2/M transition was mediated by Cyclin A, Cyclin B1 (161), CDK1 (162, 163), PLK1 (128), Ki67, PRC1 (164) and CDC25A/B (165) (sustained proliferative signaling). Additional to developing insensitivity to growth signal inhibitors (Cip, Kip proteins), FOXM1 could also keep proliferative signals like TGFα (166), JNK1 (154), IGF1 (167), and NEDD4-1 (168), continuously switched on (self-sufficiency in growth signals).

FOXM1 has been shown to be involved in generation of genomic instability by altered DNA damage response (CHK1) and repair (BRCA2, EXO1, RAD51, BRIP1, XRCC1/2) (169, 170). Apart from this, its role in execution of mitosis by genes such as BUBR1, NEK2 (171), Aurora A/B (172, 173), KIF4A (174), CENP-A/B/E/F, CEP55, BORA and CDCA2/8 (175) ensures high fidelity of cell division process (genome instability and mutation).

Supplemental to uncontrolled proliferation and development of a full blown cancer is resisting cell death. Affirming to its role in cancer development and progression, FOXM1 inhibits apoptosis by BCL2 (176), XIAP, survivin (177) and enhance autophagy by LC3 and Beclin1 (178). Moreover, FOXM1 was shown to avoid senescence by activation of BMI1 and ROS scavengers’ catalase, MnSOD1, and PRDX3 (179) (evading apoptosis). It has been found that FOXM1 depletion sensitizes the cells to premature senescence thus slowing down cancer progression. Overcoming replicative senescence by activation of hTERT mediated telomeric activity further established FOXM1 as an oncogene (limitless replicative potential).

Another hallmark of cancer is the reprogramming of energy metabolism by aerobic glycolysis. FOXM1 promotes this by direct activation of LDHA (180), IDH1 (181), GLUT1 and HK2 (182). FOXM1 is also implicated in regulating the expression of pentatricopeptide repeat domain 1 (PTCD1), a mitochondrial leucine-specific tRNA binding protein which inhibits oxidative phosphorylation (183) (deregulating cellular energetics). In order to maintain a constant nutrient supply for this energy metabolism altered cells, FOXM1 stimulated neovascularization by promoting expression of FGF9, VEGF/VEGFR triggered by HIF/ROS signaling (184) (sustained angiogenesis). Epithelial-mesenchymal transition events lead to the escape of cells from primary site to enable cancer progression. FOXM1 has been shown to facilitate this escape by activating invasion and migration (MMP2, MMP9 (185), uPA, UPAR (186), LOX, LOXL2 (187), ROCK1 (8), SNAIL (188), eEF2K (189), E-Cadherin (190)) (tissue invasion and metastasis).

Recent developments have revealed the role of stemness in sustaining cancer contrary to its physiological functions. They present with enhanced capacities for self-renewal, proliferation, differentiation, metastasis, homing and drug resistance. FOXM1 has been demonstrated to promote these functions of stemness through regulation of OCT4, NANOG and SOX2 (191). Drug resistance phenotypes in cancer cells have been gained by the action of FOXM1 regulating the expression of ABCB1 (192), ABCC4 (193), ABCC5 (194), NBS1 (195) and BRIP1 (169). **Figure 6** summarizes the downstream factors of FOXM1 in mediating Hallmarks of cancer.

In addition to the regulation of cancer by these various transcriptional mechanisms, FOXM1 has been shown to exert its role in cancer development by several critical protein-protein interactions. FOXM1 interaction with MELK, PIN1 and pSTAT3 induced neurosphere development, BRAFV600E stimulated melanoma progression and radioresistance in glioblastoma respectively (130, 196, 197). FOXM1-SMAD interaction occurs downstream of the TGFβ signaling pathway and promotes tumor progression (198). Nucleophosmin (NPN) was shown to sustain FOXM1 nuclear localization in cancer cells, whose mutation in AML interestingly led to FOXM1 inactivation by cytoplasmic shuttling (199). Binding with β-catenin and NFkB in CML led to development of self-renewal capacity and enhanced survival (200). Although FOXM1 is a master transcriptional factor, it was shown to be a cofactor of β-catenin in regulating Wnt signaling mediated tumorigensis in glioma. FOXM1 was also found to be stabilized by its interaction with PHGDH, which results in proliferation, invasion and tumorigenesis (201). Additionally, FOXM1 was also shown to interact with lncRNA, PVT1 and promote tumor growth and metastasis in gastric cancer (116). Heat shock protein 70 (HSP70) was shown to be a direct biological inhibitor of FOXM1 by affecting its transactivation capabilities (202). **Table 2** lists various interacting molecules of FOXM1.

**Table 2:** List of various interacting partners of Forkhead transcription factor M1 (FOXM1).

Sl. No.	Interactors	Function	Physiological context
1.	APC^cdh1^, cdc27 subunit	Degradation of FOXM1	Cell proliferation (144)
2.	β-catenin	The interaction with FOXM1 leads to its nuclear translocation.	Proliferation, invasion and migration, control Wnt target gene expression (200)
3.	CDC25A	Interaction leads to activation FOXM1 transcriptional activity.	Regulate cell cycle (156)
4.	EPS8	Partnering factor in regulating G2/M progression.	Proliferation and migration/invasion (46)
5.	FBXO31	Destabilize FOXMI by promoting degradation	Genomic instability (145)
6.	FBXW7	Stabilize FOXM1 by deubiquitination.	Cell proliferation, invasion, apoptosis (151) (203),
7.	GSK3A	Phosphorylate FOXM1. Wnt signaling hampers this process.	Cell proliferation, invasion, apoptosis (151) (203),
8.	HIPK2	Phosphorylation of FOXM1	Cell proliferation (133)
9.	HSP 70	Interact with FOXM1 during proteotoxic stress and inhibit FOXM1’s DNA binding ability.	Provide resistance to cell death and chemotherapeutics (202)
10.	MELK	Phosphorylate FOXM1 and increase its transcriptional activity	Cell cycle progression (130)
11.	MTDH	Stabilization of FOXM1 and also increase its transcriptional activity	Cell proliferation, angiogenesis and invasion (204)
12.	NPM	Helps FOXM1 to localize in the nucleus in cancer cells	Cell proliferation (199), drug resistance (205).
13.	OTUB1	Suppress FOXM1 degradation by deubiquitination.	Cell proliferation (206, 207)
14.	P19ARF	Inhibit FOXM1 transcriptional activity	Cell proliferation (208)
15.	PHGDH	Stabilization of FOXM1	Proliferation, invasion (201)
16.	PIN1	Increase FOXM1 activity.	Proliferation, metastasis and drug resistance (196)
17.	PLK1	Increase transactivation capability of FOXM1 by phosphorylation.	Cell cycle progression (128)
18.		Phosphorylate FOXM1 and increase its transcriptional activity and aids mitotic progression.	Cell proliferation (128)
19.	PP2A/B55α	Dephosphorylate FOXM1 and negatively regulates FoxM1 activity	Cell proliferation
20.	RB1	Interaction with RB1 represses FOXM1. RB1 and DNMT3b interaction makes FOXM1 to function as repressor for some genes	Differentiation of luminal epithelial progenitors (209) (210)
21.	RNF168 RNF8	Ubiquitination and degradation of SUMOylated FOXM1.	Cell proliferation (150)
22.	SIRT1	Destabilize FOXM1 by activating APC cdh1 mediated degradation	Cell proliferation (136)
23.	SMAD 3	Its interaction with FOXM1 sustain SMAD3/SMAD4 complex activation	Metastasis (198)
24.	SP1	FOXM1 and SP1 interaction induce EGF dependent COX2 expression.	Cell proliferation (211)
25.	STAT3	FOXM1 interacts with FOXM1 following radiation treatment	Confers radio resistance (197)
26.	SUMO 1	Destabilization/Activation	Proliferation, invasion, metastasis (138) (141),
27.	USP21	Stabilize FOXM1 expression by deubiquitination.	Proliferation, Drug sensitivity (148).
28.	USP5	Stabilization of FOXM1	Proliferation, invasion, migration (147)

Role of FOXM1 interacting proteins and their physiological effects in context of cancer.

## Pharmacological Inhibitors of FOXM1

In general, activity of a protein is regulated by effectors ranging from ions to large macromolecules. FOXM1 expression and activity are tightly controlled by several inbuilt cellular mechanisms which include autoinhibition of N-terminal repressor domain, miRNAs and regulation by proteins like p19ARF, p53, RB, KLF4 and FOXO3. FOXM1 is overexpressed in most cancers and is also known to have implications in all hallmarks of cancer, primarily based on its ability to transcriptionally activate several downstream effectors. This necessitated the development of novel chemical interventions which have been significantly progressing over the last decade. Indirect targeting of FOXM1 have been established using inhibitors of upstream factors like PIN1 (DRI peptides), SP1 (Thiazolidinediones) and GLI1 (Diarylheptanoids) (196, 212, 213). Traditional medicinal derivatives like Honokiol and Casticin have also shown to supress FOXM1 and thereby its target genes (214, 215). Druggability of transcription factors has to account for any undesired effects due to the wide array of processes regulated by it. Therefore, specific inhibitors like DRI and 9R-201 peptides, FOXM1 Aptamer and TFI-10 that bind to FOXM1 are more effective in reducing tumor growth and activating apoptotic pathways (196, 216, 217). Additionally, several proteasome inhibitors like MG132, Brotezomib (218, 219) and thiazol antibiotics have been shown to significantly reduce FOXM1 expression. Siomycin a and Thiostrepton have been widely used across various cancer cell lines due to its specificity to inhibit FOXM1 by interacting with the DNA binding domain and preventing any auto feedback *via* NFRM loop (220). Despite development of these inhibitors, their clinical outcomes have been limited due to several kinetic and clinical factors. High throughput screening and phage library preparation have led to the designing of specific inhibitors like 9R-201, FOXM1 Apt, FDI-6 and RCM1 (221, 222) that primarily bind to the FOXM1 DBD *via* an electron deficient sulfur atom (π-sulfur) to His-287 in the protein. Although several inhibitors have been developed for targeting FOXM1 at multiple levels, specific delivery to cancer cells is the need of the hour to avoid disbalance of cellular homeostasis. Novel strategies to target FOXM1 dependent cancers could include combinatorial therapies with inclusion of chemosensitizers. A summary of the inhibitors of FOXM1 have been listed in **Table 3**.

**Table 3:** List of inhibitors of Forkhead transcription factor M1 (FOXM1).

Sl. No.	Inhibitor	General Action	Indirect/Direct (I/D) mechanism		Cancer	Cell Lines
**1.**	**Bortezomib**	Proteosome inhibitor	**I**	–	Osteosarcoma	U2OS (218)
					Pancreatic cancer	Mia PaCa-2 (219)
					Multiple myeloma	U266 and RPMI8226 (218)
					Leukemia	HL-60 (218)
**2.**	**Casticin**	Anti-malarial sensitizer	**I**	FOXO3a-FOXM1 (Inhibit FOXO3a)	Breast cancer	MDA-MB231, MCF-7 (223)
					Ovarian cancer	SKOV3, A2780 (215)
					Liver cancer	HEPG2, PLC/PRF/5 (224)
**3.**	**Daunorubicin**	Topoisomerase inhibitor	**I**	p53-p21-FOXM1	Lung cancer	H1299 (225)
					Breast cancer	MCF-7 (225)
					Liver cancer	HepG2 (225)
**4.**	**Diarylheptanoids**	Anti-oxidants	**I**	Shh-Gli-FOXM1 (Inhibit Gli1)	Pancreatic cancer	PANC-1 (213)
**5.**	**DFOG**	Genistein derivative	**I**	–	Ovarian cancer	CoC1, SKOV3 (226)
					Gastric cancer	AGS, SGC-7901 (227)
**6.**	**DIM**	Radioprotector	**I**	–	Breast cancer	MDA-MB231, MDA-MB468, SKBR3, MCF-7 (228)
					Colorectal cancer	DLD-1 and HCT116 (229)
					Gastric cancer	SNU638 (230)
**7.**	**FDI-6**	FOXM1 specific drug	**D**	Blocks FOXM1 DBD	Breast cancer	MCF-7, MDA-MB231 (231)
					Laryngeal Cancer	Hep-2 (221)
**8.**	**FOXM1 Apt**	FOXM1-specific single stranded DNA aptamer generated by SELEX	**D**	Target FOXM1 DBD	Breast cancer	MDA-MB436 (217)
**9.**	**Fulvestrant (ICI182780)**	Estrogen receptor antagonist	**I**	ERβ1/ERα-FOXM1 axis (Inhibits ERα)	Breast cancer	MCF-7, ZR-75-1 (35)
**10.**	**Genistein**	Angiogenesis inhibitor	**I**	–	Pancreatic cancer	BxPC-3, HPAC, MIAPaCa-2, PANC28 (232)
					Lung cancer	IMR-90, H460, A549, H446 (233)
**11.**	**Honokiol**	Anti-inflammatory, anti-oxidant	**D**	Interact with FOXM1	Osteosarcoma	U2OS C3 (214)
					Prostate cancer	DU145 (214)
					Breast cancer	MDA-MB231 (214)
**12.**	**MG132**	Proteosome inhibitor	**I**	–	Pancreatic cancer	Mia PaCa-2 (219)
					Breast cancer	MDA-MB231 (219)
					Colorectal cancer	HCT-116 (219)
**13.**	**Mithramycn A**	DNA binding neuroprotective antibiotic	**I**	Sp1-FOXM1 (Inhibit Sp1)	Liver cancer	HEPG2, PLC/PRF/5 (212)
**14.**	**Monensin**	Polyether antibiotic	**D**	Interact with FOXM1 DBD	Prostate cancer	ENZ^R^-CRPC (234)
**15.**	**Natura-α**	STAT3 inhibitor	**I**	–	Prostate cancer	LNCaP, LNCaP-AI, PC-3, and DU145 (235)
**16.**	**Nutlin-3**	MDM2-p53 interaction inhibitor	**I**	MDM2-p53-FOXM1 (MDM2 inhibitor)	Osteosarcoma	U2OS (225), OVCAR10
					Lung cancer	NCI-H23
					Ovarian cancer	A2780
					Colorectal cancer	HCT-116 (225)
**17.**	**Panepoxydone**	Fungal NFkB pathway inhibitor	**I**	–	Breast cancer	MCF7, MDA-MB231 (236)
**18.**	**Peptide 9R-P201**	High affinity peptides against FOXM1C from the phage random library	**D**	Target FOXM1 DBD	Liver cancer	HepG2 (216)
**19.**	**RCM-1**	FOXM1 drug identified by high throughput screen	**I**	Increase ubiquitination	Osteosarcoma	U2OS C3
					Melanoma	B16-F10 (mouse) (222)
					Prostate cancer	MyC-CaP (mouse) (222)
					Breast cancer	4T1 (mouse) (222)
					Lung cancer	A549 (222)
**20.**	**S331/704 DRI peptide**	ATP competitive-BRAF Kinase	**I**	Pin1-FOXM1 binding	Melanoma	Colo829, Malme-3M (196)
**21.**	**Siomycin A**	Thiazole antibiotic	**D**	Interact with FOXM1 DBD and by NFRM loop	Liver cancer	Huh7, Hep3B, SK-Hep (220)
					Lung cancer	A549 (220)
					Colorectal cancer	SW480, SW620 (220)
					Melanoma	DM443, DM366, DM833, DM646 (237)
					Leukemia	CEM, HL60, U937 (220)
**22.**	**Thiazolidinediones**	Anti-hyperglycemic	**I**	Sp1-FOXM1 (Inhibit Sp1)	Liver cancer	HepG2, PLC/PRF/5 (212)
**23.**	**Thiostrepton**	Thiazole antibiotic	**D**	Interact with FOXM1 DBD and by NFRM loop	Breast cancer	MCF-7, MDA-MB436, MDA-MB231
					Ovarian cancer	SKOB3, OVCAR3 (238)
					Colorectal cancer	HCT-15, HT-29 (239)
					Melanoma	DM443, DM366, DM833, DM646 (237)
					Leukemia	MV4-11, THP1, CEM, HL60, U937
					Laryngeal Squamous Ca	Hep-2
					Thyroid cancer	BCPAP, TCP-1
**24.**	**TFI-10**	Modified Thiazolidinediones	**D**	Interact with FOXM1 DBD	Breast cancer	MDA-MB231 (240)
**25.**	**TMPP**	IER5 activator	**I**	IER5-FOXM1 axis	Leukemia	U937, YRK2 (241)
**26.**	**U0126**	MEK1/2 inhibitor	**I**	MEK-ERK-FOXM1 axis	Ovarian cancer Breast cancer	OVCA433, A2780cp (242) MCF-7, ZR-75-30 (243)
**27.**	**Ursolic acid**	Anti-inflammatory, Anti-apoptotic	**I**	–	Breast cancer	MCF-7 (244)
**28.**	**Vemurafenib (PLX4032)**	BRAF inhibitor	**I**	BRAF-ERK-FOXM1-AuroraB	Melanoma	A375, 501mel (173)

Chemical inhibitors of FOXM1 and their mechanism of action, tested in respective cell lines. TMPP, 2,3,4-tribromo-3-methyl-1-phenylphospholane 1-oxide; DIM, 3,3’-diindolylmethane; DFOG, 7-difluoromethoxyl-5,4’-di-n-octyl-genistein.

## Conclusion and Future Perspective

In this review, we have addressed various regulatory pathways and mechanisms by which FOXM1 is altered and its implications in modulating different hallmarks of cancer. Based on its role in controlling various aspects of cellular processes as discussed throughout this review, FOXM1 is reinforced as an oncogene. We have elaborately discussed several signaling pathways that are deregulated in cancer. RAS signaling pathway increase FOXM1 expression and also its nuclear translocation. FOXM1 further keep this signaling switched on through the inhibition of RASSF1A tumor suppressor protein. OPN activated α6-integrin leads to a triggering of FOXM1-FGFR/MET loop that may culminate in metastasis. Additionally, activation of hedgehog signals *via* Gli1 correlate with FOXM1 expression and predict metastatic outcomes. We also emphasized the importance of Vitamin D in FOXM1 regulation, as dietary variations are an emerging area in cancer research.

Altered expression of FOXM1 transcription factor has been seen in majority of the cancers. The deregulation of FOXM1 observed during tumorigenesis may be traced back to its own miRNAs. miRNAs are categorized either as oncogenic or tumor suppressive based on their expression pattern in malignancy. They impart their effect through direct association with FOXM1 or indirectly through its regulatory factors. Identification of FOXM1 miRNAs as tumor biomarkers has necessitated the need for further studies on miRNAs regulation.

Hampered post-translational modifications like SUMOylation by infectious viral particles or resistance to chemotherapeutic drugs tactically modulate FOXM1 function leading to a growth advantage. However a word of caution here is that predicting the eventual direction of FOXM1 function in cancer progression would be dependent on elucidating the SUMOylation fate determining factors. Sustained FOXM1 activity in cancer may be promoted by deregulated expression of various kinases such as pERK as seen in high grade ovarian cancer (245). Existence of a positive feedback loop between FOXM1 and kinases such as PLK1 and CDK1 further support a sustained FOXM1 activation in various malignancies.

**Figure 7:**
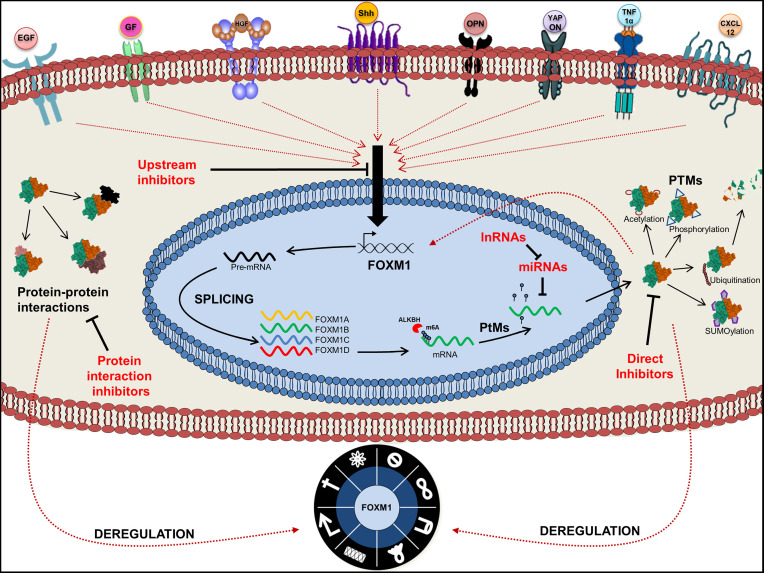
Graphical abstract of Forkhead transcription factor M1 (FOXM1) regulation. Pictorial represents upstream regulators of FOXM1 and its various fates. Point of action of FOXM1 chemical inhibitors is also indicated.

Despite enormous amount of literature describing the various processes that associate FOXMI with cancer, its potential as a target molecule for cancer treatment has been limited by the fact that it is a transcription factor. FOXM1 inhibition by siRNA or chemical inhibitors has shown to reduce the tumor size and also sensitized the tumor cells to chemotherapeutic agents. We envisage that developing targeted therapy by identifying interacting partners, pathways activated and various crosstalk mechanisms is the way forward in the quest for discovering inhibitors to halt the march of cancer. Furthermore, FOXM1 acts as a barrier for most of the existing chemotherapeutic regimes as evidenced from the drug resistance to Herceptin and taxol, mediated by degradation of p27 and *via* stathmin respectively (246, 247). FOXM1 expression and its modulation by various mechanisms in response to different cues have necessitated a thorough understanding of signaling molecules, various post transcriptional and translational mechanisms. This will pave the way for devising precise therapeutic target points of FOXM1 dependent cancer initiation, progression, genomic instability and cancer cell drug resistance processes. Graphical abstract of the review has been represented in **Figure 7**.

## Author Contributions

DK, SJ, AN wrote the manuscript. DK, SJ designed the figures. AN provided guidance and revised the manuscript. All authors contributed to the article and approved the submitted version.

## Conflict of Interest

The authors declare that the research was conducted in the absence of any commercial or financial relationships that could be construed as a potential conflict of interest.

## Abbreviations

ANO2, Anoctamin 2; CTNNBL1, Catenin Beta Like 1; DCP1B, Decapping mRNA 1B; DOT1L, DOT1 Like Histone Lysine Methyltransferase; ECT2, Epithelial Cell Transforming 2; EPS8, Epidermal Growth Factor Receptor Pathway Substrate 8; FBX, F-Box and WD repeat Domain; FOXM1, Forkhead transcription factor M1; KLF, Kruppel Like Factor; METTL3, Methyltransferase Like 3; MTSS1, Metastasis Suppressor 1; OTUB1, OTU Deubiquitinase, Ubiquitin Aldehyde Binding 1; PBD, PLK1 Binding Domain; PDA, Pancreatic Adenocarcinoma; PQC, Protein Quality Control; RASSF1A, Ras Association Domain Family Member 1; RNF, Ring Finger Protein; RTK, Receptor Tyrosine Kinase; SETD3, SET Domain Containing 3, Actin Histidine Methyltransferase; SLC2A14, Solute Carrier Family 2 Member 14; SRp20, Serine and Arginine rich Splicing factor; STUbLs, SUMO-targeted Ubiquitin Ligases; TAD, Transcriptional Activation domain; TCGA, The Cancer Genome Atlas Program; UCA1, Urothelial Cancer Associated 1; UCHL3, Ubiquitin C-Terminal Hydrolase L3; USP, Ubiquitin Specific Peptidase.

## References

[1] Weigel D Jürgens G Küttner F Seifert E Jäckle H. The homeotic gene fork head encodes a nuclear protein and is expressed in the terminal regions of the Drosophila embryo. Cell (1989) 57:645–58. 10.1016/0092-8674(89)90133-5 2566386

[2] Marsden I Chen Y Jin C Liao X. Evidence that the DNA binding specificity of winged helix proteins is mediated by a structural change in the amino acid sequence adjacent to the principal DNA binding helix. Biochemistry (1997) 36:13248–55. 10.1021/bi971514m 9341214

[3] Hannenhalli S Kaestner KH. The evolution of Fox genes and their role in development and disease. Nat Rev Genet (2009) 10:233–40. 10.1038/nrg2523 PMC2733165 19274050

[4] Kaestner KH Knöchel W Martínez DE Kno W Martı DE. Unified nomenclature for the winged helix / forkhead transcription factors. Genes Dev (2000) 14:142–6. 10.1101/gad.14.2.142 10702024

[5] Golson ML Kaestner KH. Fox transcription factors: From development to disease. Dev (2016) 143:4558–70. 10.1242/dev.112672 PMC5201025 27965437

[6] Stott SRW Ang SL. The Generation of Midbrain Dopaminergic Neurons. In: JL Rubenstein L John P Rakic (ed.) Comprehensive Developmental Neuroscience: Patterning and Cell Type Specification in the Developing CNS and PNS. Amsterdam: Elsevier (2013) vol. 1, pp. 435–48.

[7] Korver W Roose J Heinen K Olde Weghuis D de Bruijn D Geurts van Kessel A. The Human TRIDENT/HFH-11/FKHL16 Gene: Structure, Localization, and Promoter Characterization. Genomics (1997) 46:435–42. 10.1006/geno.1997.5065 9441747

[8] Zhang X Zhang L Du Y Zheng H Zhang P Sun Y. A novel FOXM1 isoform, FOXM1D, promotes epithelial-mesenchymal transition and metastasis through ROCKs activation in colorectal cancer. Oncogene (2017) 36:807–19. 10.1038/onc.2016.249 PMC5311249 27399334

[9] Ye H Kelly TF Samadani U Lim L Rubio S Overdier DG. Hepatocyte Nuclear Factor 3/fork head Homolog 11 Is Expressed in Proliferating Epithelial and Mesenchymal Cells of Embryonic and Adult Tissues. Mol Cell Biol (1997) 17:1626–41. 10.1128/mcb.17.3.1626 PMC231888 9032290

[10] Koo C-Y Muir KW W-F. Lam E. FOXM1: From cancer initiation to progression and treatment. BBA - Gene Regul Mech (2012) 1819:28–37. 10.1016/j.bbagrm.2011.09.004 21978825

[11] Li Y Wu F Tan Q Guo M Ma P Wang X. The multifaceted roles of FOXM1 in pulmonary disease. Cell Commun Signal (2019) 17:1–16. 10.1186/s12964-019-0347-1 30992007 PMC6469073

[12] Nandi D Cheema PS Jaiswal N Nag A. FoxM1: Repurposing an oncogene as a biomarker. Semin Cancer Biol (2018) 52:74–84. 10.1016/j.semcancer.2017.08.009 28855104

[13] Halasi M Gartel AL. FOX(M1) News–It Is Cancer. Mol Cancer Ther (2013) 12:245–54. 10.1158/1535-7163.MCT-12-0712 PMC3596487 23443798

[14] Tate JG Bamford S Jubb HC Sondka Z Beare DM Bindal N. COSMIC: The Catalogue Of Somatic Mutations In Cancer. Nucleic Acids Res (2019) 47:D941–7. 10.1093/nar/gky1015 PMC6323903 30371878

[15] Yu J Deshmukh H Payton JE Dunham C Scheithauer BW Tihan T. Array-based comparative genomic hybridization identifies CDK4 and FOXM1 alterations as independent predictors of survival in malignant peripheral nerve sheath tumor. Clin Cancer Res (2011) 17:1924–34. 10.1158/1078-0432.CCR-10-1551 21325289

[16] Hu Z Mao J-H Curtis C Huang G Gu S Heiser L. Genome co-amplification upregulates a mitotic gene network activity that predicts outcome and response to mitotic protein inhibitors in breast cancer. Breast Cancer Res (2016) 18:70. 10.1186/s13058-016-0728-y 27368372 PMC4930593

[17] Barger CJ Branick C Chee L Karpf AR. Pan-cancer analyses reveal genomic features of FOXM1 overexpression in cancer. Cancers (Basel) (2019) 11:E251. 10.3390/cancers11020251 30795624 PMC6406812

[18] Barger CJ Zhang W Hillman J Stablewski AB Higgins MJ Vanderhyden BC. Genetic determinants of FOXM1 overexpression in epithelial ovarian cancer and functional contribution to cell cycle progression. Oncotarget (2015) 6:27613–27. 10.18632/oncotarget.4546 PMC4695012 26243836

[19] Kim P Zhou X. FusionGDB: Fusion gene annotation DataBase. Nucleic Acids Res (2019) 47:D994–D1004. 10.1093/nar/gky1067 30407583 PMC6323909

[20] Chai YJ Yi JW Oh SW Kim YA Yi KH Kim JH. Upregulation of SLC2 (GLUT) family genes is related to poor survival outcomes in papillar thyroid carcinoma: Analysis of data from The Cancer Genome Atlas. Surgery (2017) 161(1)188–94. 10.1016/j.surg.2016.04.050 27842912

[21] Hoff AM Alagaratnam S Zhao S Bruun J Andrews PW Lothe RA. Identification of novel fusion genes in testicular germ cell tumors. Cancer Res (2016) 76:108–16. 10.1158/0008-5472.CAN-15-1790 PMC4704135 26659575

[22] Zhang X Zeng J Zhou M Li B Zhang Y Huang T. The tumor suppressive role of miRNA-370 by targeting FoxM1 in acute myeloid leukemia. Mol Cancer (2012) 11:56. 10.1186/1476-4598-11-56 22900969 PMC3533721

[23] Shi M Cui J Xie K. Signaling of miRNAs-FOXM1 in cancer and potential targeted therapy. Curr Drug Targets (2013) 14:1192–202. 10.2174/13894501113149990192 PMC4081534 23834153

[24] Kopanja D Pandey A Kiefer M Wang Z Chandan N Carr JR. Essential roles of FoxM1 in Ras-induced liver cancer progression and in cancer cells with stem cell features. J Hepatol (2015) 63(2):429–36. 10.1016/j.jhep.2015.03.023 PMC4508215 25828473

[25] Blanchard TG Czinn SJ Banerjee V Sharda N Bafford AC Mubariz F. Identification of cross talk between FoxM1 and RASSF1A as a therapeutic target of colon cancer. Cancers (Basel) (2019) 11:199. 10.3390/cancers11020199 PMC6406751 30744076

[26] Hesson LB Cooper WN Latif F. The role of RASSF1A methylation in cancer. Dis Markers (2007) 23(1-2):73–87. 10.1155/2007/291538 17325427 PMC3850810

[27] Schagdarsurengin U Wilkens L Steinemann D Flemming P Kreipe HH Pfeifer GP. Frequent epigenetic inactivation of the RASSF1A gene in hepatocellular carcinoma. Oncogene (2003) 22:1866–71. 10.1038/sj.onc.1206338 12660822

[28] Hu L Chen G Yu H Qiu X. Clinicopathological significance of RASSF1A reduced expression and hypermethylation in hepatocellular carcinoma. Hepatol Int (2010) 4:423–32. 10.1007/s12072-010-9164-8 PMC2836437 20305761

[29] Cui J Xia T Xie D Gao Y Jia Z Wei D. HGF/Met and FOXM1 form a positive feedback loop and render pancreatic cancer cells resistance to Met inhibition and aggressive phenotypes. Nat Publ Gr (2016) 35:4708–18. 10.1038/onc.2016.14 PMC4985506 26876216

[30] Francica P Nisa L Aebersold DM Langer R Bladt F Blaukat A. Depletion of FOXM1 via MET targeting underlies establishment of a DNA damage-induced senescence program in gastric cancer. Clin Cancer Res (2016) 22:5322–36. 10.1158/1078-0432.CCR-15-2987 27185371

[31] Hsieh CH Chu CY Lin SE Yang YCSH Chang HS Yen Y. Tesc promotes tgf-α/egfr-foxm1-mediated tumor progression in cholangiocarcinoma. Cancers (Basel) (2020) 12(5):1105. 10.3390/cancers12051105 PMC7281536 32365487

[32] Francis RE Myatt SS Krol J Hartman J Peck B McGovern UB. FoxM1 is a downstream target and marker of HER2 overexpression in breast cancer. Int J Oncol (2009) 35:57–68. 10.3892/ijo_00000313 19513552 PMC3065068

[33] Bektas N Haaf AT Veeck J Wild PJ Lüscher-Firzlaff J Hartmann A. Tight correlation between expression of the Forkhead transcription factor FOXM1 and HER2 in human breast cancer. BMC Cancer (2008) 8:42. 10.1186/1471-2407-8-42 18254960 PMC2265720

[34] Ahn H Sim J Abdul R Chung MS Paik SS Oh Y-H. Increased Expression of Forkhead Box M1 Is Associated with Aggressive Phenotype and Poor Prognosis in Estrogen Receptor-Positive Breast Cancer. J Korean Med Sci (2015) 30:390–7. 10.3346/jkms.2015.30.4.390 PMC4366959 25829806

[35] Millour J Constantinidou D Stavropoulou AV Wilson MSC Myatt SS Kwok JMM. FOXM1 is a transcriptional target of ERα and has a critical role in breast cancer endocrine sensitivity and resistance. Oncogene (2010) 29:2983–95. 10.1038/onc.2010.47 PMC2874720 20208560

[36] Lu M Hu K. Correlation of HER2 and FOXM1 in human colorectal carcinoma and its clinical significance. Int J Clin Pathol (2017) 10(12):11624–34. PMC6966020 31966520

[37] de Olano N Koo C-Y Monteiro LJ Pinto PH Gomes AR Aligue R. The p38 MAPK-MK2 axis regulates E2F1 and FOXM1 expression after epirubicin treatment. Mol Cancer Res (2012) 10:1189–202. 10.1158/1541-7786.MCR-11-0559 PMC3447739 22802261

[38] Millour J de Olano N Horimoto Y Monteiro LJ Langer JK Aligue R. ATM and p53 regulate FOXM1 expression via E2F in breast cancer epirubicin treatment and resistance. Mol Cancer Ther (2011) 10:1046–58. 10.1158/1535-7163.MCT-11-0024 PMC4845881 21518729

[39] Yan D Yan X Dai X Chen L Sun L Li T. Activation of AKT/AP1/FoxM1 signaling confers sorafenib resistance to liver cancer cells. Oncol Rep (2019) 42:785–96. 10.3892/or.2019.7192 31233189

[40] Gouazé-Andersson V Ghérardi M-J Lemarié A Gilhodes J Lubrano V Arnauduc F. FGFR1/FOXM1 pathway: a key regulator of glioblastoma stem cells radioresistance and a prognosis biomarker. Oncotarget (2018) 9:31637–49. 10.18632/oncotarget.25827 PMC6114977 30167084

[41] Kowalski-Chauvel A Gouaze-Andersson V Baricault L Martin E Delmas C Toulas C. Alpha6-integrin regulates FGFR1 expression through the ZEB1/YAP1 transcription complex in glioblastoma stem cells resulting in enhanced proliferation and stemness. Cancers (Basel) (2019) 11:406. 10.3390/cancers11030406 PMC6468800 30909436

[42] Cao J Li J Sun L Qin T Xiao Y Chen K. Hypoxia-driven paracrine osteopontin/integrin αvβ3 signaling promotes pancreatic cancer cell epithelial–mesenchymal transition and cancer stem cell-like properties by modulating forkhead box protein M1. Mol Oncol (2019) 13:228–45. 10.1002/1878-0261.12399 PMC6360359 30367545

[43] Xie Y Li Y Kong Y. OPN Induces FoxM1 Expression and Localization through ERK 1/2, AKT, and p38 Signaling Pathway in HEC-1A Cells. Int J Mol Sci (2014) 15:23345–58. 10.3390/ijms151223345 PMC4284770 25522167

[44] Chen C Liang Z Huang W Li X Zhou F Hu X. Eps8 regulates cellular proliferation and migration of breast cancer. Int J Oncol (2015) 46:205–14. 10.3892/ijo.2014.2710 25333707

[45] Wang H Teh M-T Ji Y Patel V Firouzabadian S Patel AA. EPS8 upregulates FOXM1 expression, enhancing cell growth and motility. Carcinogenesis (2010) 31:1132–41. 10.1093/carcin/bgq058 PMC2878363 20351091

[46] Ngan AWL Tsui MG So DHF Leung WY Chan DW Yao KM. Novel nuclear partnering role of EPS8 with FOXM1 in regulating cell proliferation. Front Oncol (2019) 9:154. 10.3389/fonc.2019.00154 30941306 PMC6433973

[47] Wang S Zhang S Li J Xu X Weng Y Zheng M. CXCL12-induced upregulation of FOXM1 expression promotes human glioblastoma cell invasion. Biochem Biophys Res Commun (2014) 447:1–6. 10.1016/j.bbrc.2013.12.079 24561124

[48] Teh M-T Wong S-T Neill GW Ghali LR Philpott MP Quinn AG. FOXM1 Is a Downstream Target of Gli1 in Basal Cell Carcinomas. Cancer Res (2002) 15;62(16):4773–80. 12183437

[49] Gialmanidis IP Bravou V Amanetopoulou SG Varakis J Kourea H Papadaki H. Overexpression of hedgehog pathway molecules and FOXM1 in non-small cell lung carcinomas. Lung Cancer (2009) 66:64–74. 10.1016/j.lungcan.2009.01.007 19200615

[50] Chen H Wang J Yang H Chen D Li P. Association between FOXM1 and hedgehog signaling pathway in human cervical carcinoma by tissue microarray analysis. Oncol Lett (2016) 12(4):2664–73. 10.3892/ol.2016.4932 PMC5038455 27698840

[51] Wang D Hu G Du Y Zhang C Lu Q Lv N. Aberrant activation of hedgehog signaling promotes cell proliferation via the transcriptional activation of forkhead Box M1 in colorectal cancer cells. J Exp Clin Cancer Res (2017) 36:23. 10.1186/s13046-017-0491-7 28148279 PMC5288899

[52] Han Y. Analysis of the role of the Hippo pathway in cancer. J Transl Med (2019) 17:1–17. 10.1186/s12967-019-1869-4 30961610 PMC6454697

[53] Pan D. Hippo signaling in organ size control. Genes Dev (2007) 21:886–97. 10.1101/gad.1536007 17437995

[54] Mizuno T Murakami H Fujii M Ishiguro F Tanaka I Kondo Y. YAP induces malignant mesothelioma cell proliferation by upregulating transcription of cell cycle-promoting genes. Oncogene (2012) 31:5117–22. 10.1038/onc.2012.5 22286761

[55] Eisinger-Mathason TSK Mucaj V Biju KM Nakazawa MS Gohil M Cash TP. Deregulation of the Hippo pathway in soft-tissue sarcoma promotes FOXM1 expression and tumorigenesis. Proc Natl Acad Sci U S A (2015) 112:E3402–11. 10.1073/pnas.1420005112 PMC4491775 26080399

[56] Rosenfeldt HM Amrani Y Watterson KR Murthy KS Panettieri RA Spiegel S. Sphingosine-1-phosphate stimulates contraction of human airway smooth muscle cells. FASEB J (2003) 17:1789–99. 10.1096/fj.02-0836com 14519658

[57] Weiler SME Pinna F Wolf T Lutz T Geldiyev A Sticht C. Induction of Chromosome Instability by Activation of Yes-Associated Protein and Forkhead Box M1 in Liver Cancer. Gastroenterology (2017) 152:2037–51.e22. 10.1053/j.gastro.2017.02.018 28249813

[58] Sun H-L Men J-R Liu H-Y Liu M-Y Zhang H-S. FOXM1 facilitates breast cancer cell stemness and migration in YAP1-dependent manner. Arch Biochem Biophys (2020) 685:108349. 10.1016/j.abb.2020.108349 32209309

[59] Xia L Mo P Huang W Zhang L Wang Y Zhu H. The TNF-α/ROS/HIF-1-induced upregulation of foxMI expression promotes HCC proliferation and resistance to apoptosis. Carcinogenesis (2012) 33:2250–9. 10.1093/carcin/bgs249 22831955

[60] Garland CF Garland FC Gorham ED Lipkin M Newmark H Mohr SB. The role of vitamin D in cancer prevention. Am J Public Health (2006) 96:252–61. 10.2105/AJPH.2004.045260 PMC1470481 16380576

[61] Li Z Jia Z Gao Y Xie D Wei D Cui J. Activation of vitamin D receptor signaling downregulates the expression of nuclear FOXM1 protein and suppresses pancreatic cancer cell stemness. Clin Cancer Res (2015) 21:844–53. 10.1158/1078-0432.CCR-14-2437 PMC4334671 25501129

[62] Corbo C Orrù S Salvatore F. SRp20: An overview of its role in human diseases. Biochem Biophys Res Commun (2013) 436:1–5. 10.1016/j.bbrc.2013.05.027 23685143

[63] Jia R Li C McCoy JP Deng C-X Zheng Z-M. SRp20 is a proto-oncogene critical for cell proliferation and tumor induction and maintenance. Int J Biol Sci (2010) 6:806–26. 10.7150/ijbs.6.806 PMC3005347 21179588

[64] YUAN X SUN X SHI X JIANG C YU D ZHANG W. USP39 promotes the growth of human hepatocellular carcinoma in vitro and in vivo. Oncol Rep (2015) 34:823–32. 10.3892/or.2015.4065 26081192

[65] Yuan X Sun X Shi X Jiang C Yu D Zhang W. USP39 regulates the growth of SMMC-7721 cells via FoxM1. Exp Ther Med (2017) 13:1506–13. 10.3892/etm.2017.4115 PMC5377580 28413501

[66] Li Y Guo H Jin C Qiu C Gao M Zhang L. Spliceosome-associated factor CTNNBL1 promotes proliferation and invasion in ovarian cancer. Exp Cell Res (2017) 357:124–34. 10.1016/j.yexcr.2017.05.008 28501461

[67] Liu N Pan T. N6-methyladenosine–encoded epitranscriptomics. Nat Struct Mol Biol (2016) 23:98–102. 10.1038/nsmb.3162 26840897

[68] Wang J Wang J Gu Q Ma Y Yang Y Zhu J. The biological function of m6A demethylase ALKBH5 and its role in human disease. Cancer Cell Int (2020) 20:1–7. 10.1186/s12935-020-01450-1 32742194 PMC7388453

[69] Zhang S Zhao BS Zhou A Lin K Zheng S Lu Z. m 6 A Demethylase ALKBH5 Maintains Tumorigenicity of Glioblastoma Stem-like Cells by Sustaining FOXM1 Expression and Cell Proliferation Program. Cancer Cell (2017) 31:591–606.e6. 10.1016/j.ccell.2017.02.013 28344040 PMC5427719

[70] Davis BN Hata A. Regulation of MicroRNA Biogenesis: A miRiad of mechanisms. Cell Commun Signal (2009) 7:18. 10.1186/1478-811X-7-18 19664273 PMC3224893

[71] Catalanotto C Cogoni C Zardo G. MicroRNA in Control of Gene Expression: An Overview of Nuclear Functions. Int J Mol Sci (2016) 17(10):1712. 10.3390/ijms17101712 PMC5085744 27754357

[72] Duan N Hu X Yang X Cheng H Zhang W. MicroRNA-370 directly targets FOXM1 to inhibit cell growth and metastasis in osteosarcoma cells. Int J Clin Exp Pathol (2015) 8:10250–60. PMC4637548 26617733

[73] Feng Y Wang L Zeng J Shen L Liang X Yu H. FoxM1 is Overexpressed in Helicobacter pylori–Induced Gastric Carcinogenesis and Is Negatively Regulated by miR-370. Mol Cancer Res (2013) 11(8):834–44. 10.1158/1541-7786.MCR-13-0007 23576572

[74] Yuan Y Wang Q Cao F Han B Xu L. MiRNA-134 suppresses esophageal squamous cell carcinoma progression by targeting FOXM1. Int J Clin Exp Pathol (2019) 12:2130–8. PMC6949631 31934035

[75] Li J Chen Y Jin M Wang J Li S Chen Z. MicroRNA-134 reverses multidrug resistance in human lung adenocarcinoma cells by targeting FOXM1. Oncol Lett (2017) 13:1451–5. 10.3892/ol.2017.5574 PMC5403496 28454276

[76] Li J Wang Y Luo J Fu Z Ying J Yu Y. miR-134 inhibits epithelial to mesenchymal transition by targeting FOXM1 in non-small cell lung cancer cells. FEBS Lett (2012) 586:3761–5. 10.1016/j.febslet.2012.09.016 23010597

[77] Wei Y Wang Z Zong Y Deng D Chen P Lu J. LncRNA MFI2-AS1 promotes HCC progression and metastasis by acting as a competing endogenous RNA of miR-134 to upregulate FOXM1 expression. BioMed Pharmacother (2020) 125:109890. 10.1016/j.biopha.2020.109890 32106369

[78] Mazzu YZ Yoshikawa Y Nandakumar S Chakraborty G Armenia J Jehane LE. Methylation-associated miR-193b silencing activates master drivers of aggressive prostate cancer. Mol Oncol (2019) 13(9):1944–58. 10.1002/1878-0261.12536 PMC6717747 31225930

[79] Li L Li Z Kong X Xie D Jia Z Jiang W. Down-regulation of MicroRNA-494 via Loss of SMAD4 Increases FOXM1 and β-Catenin Signaling in Pancreatic Ductal Adenocarcinoma Cells. Gastroenterology (2014) 147:485–97.e18. 10.1053/j.gastro.2014.04.048 24859161

[80] Inoguchi S Seki N Chiyomaru T Ishihara T Matsushita R Mataki H. Tumour-suppressive *microRNA-24-1* inhibits cancer cell proliferation through targeting *FOXM1* in bladder cancer. FEBS Lett (2014) 588:3170–9. 10.1016/j.febslet.2014.06.058 24999187

[81] Sun Y Yu X Bai Q. miR-204 inhibits invasion and epithelial-mesenchymal transition by targeting FOXM1 in esophageal cancer. Int J Clin Exp Pathol (2015) 8:12775–83. PMC4680412 26722467

[82] Li T Pan H Li R. The dual regulatory role of miR-204 in cancer. Tumor Biol (2016) 37:11667–77. 10.1007/s13277-016-5144-5 PMC5080331 27438705

[83] Tan X Fu Y Chen L Lee W Lai Y Rezaei K. miR-671-5p inhibits epithelial-to-mesenchymal transition by downregulating FOXM1 expression in breast cancer. Oncotarget (2016) 7:293–307. 10.18632/oncotarget.6344 26588055 PMC4807999

[84] Tan X Li Z Ren S Rezaei K Pan Q Goldstein AT. Dynamically decreased miR-671-5p expression is associated with oncogenic transformation and radiochemoresistance in breast cancer. Breast Cancer Res (2019) 21:89. 10.1186/s13058-019-1173-5 31391072 PMC6686561

[85] He S Liao B Deng Y Su C Tuo J Liu J. MiR-216b inhibits cell proliferation by targeting FOXM1 in cervical cancer cells and is associated with better prognosis. BMC Cancer (2017) 17:673. 10.1186/s12885-017-3650-5 28978307 PMC5628450

[86] Zheng W-W Zhou J Zhang C-H Liu X-S. MicroRNA-216b is downregulated in hepatocellular carcinoma and inhibits HepG2 cell growth by targeting Forkhead box protein M1. Eur Rev Med Pharmacol Sci (2016) 20:2541–50. 27383303

[87] Yuan F Wang W. MicroRNA-802 suppresses breast cancer proliferation through downregulation of FoxM1. Mol Med Rep (2015) 12:4647–51. 10.3892/mmr.2015.3921 26080894

[88] Eissa S Matboli M Shehata HH. Breast tissue–based microRNA panel highlights microRNA-23a and selected target genes as putative biomarkers for breast cancer. Transl Res (2015) 165:417–27. 10.1016/j.trsl.2014.10.001 25445205

[89] Wang Z Zhang W Yang J Song D Wei J. Expression of miRNA-630 in bladder urothelial carcinoma and its clinical significance. J Huazhong Univ Sci Technol [Med Sci (2016) 36:705–9. 10.1007/s11596-016-1648-x 27752905

[90] Chu D Zhao Z Li Y Li J Zheng J Wang W. Increased MicroRNA-630 Expression in Gastric Cancer Is Associated with Poor Overall Survival. PloS One (2014) 9:e90526. 10.1371/journal.pone.0090526 24621930 PMC3951214

[91] Zhang J Li Y Zeng X-C Zhang T Fu B-S Yi H-M. miR-630 overexpression in hepatocellular carcinoma tissues is positively correlated with alpha-fetoprotein. Med Sci Monit (2015) 21:667–73. 10.12659/MSM.892515 PMC4356187 25731670

[92] Tang X Shi X Wang N Peng W Cheng Z. MicroRNA-215-3p Suppresses the Growth, Migration, and Invasion of Colorectal Cancer by Targeting FOXM1. Technol Cancer Res Treat (2019) 18:1533033819874776. 10.1177/1533033819874776 31607224 PMC6791039

[93] Senfter D Samadaei M Mader RM Gojo J Peyrl A Krupitza G. High impact of miRNA-4521 on FOXM1 expression in medulloblastoma. Cell Death Dis (2019) 10:696. 10.1038/s41419-019-1926-1 31541075 PMC6754377

[94] Mizoguchi A Takayama A Arai T Kawauchi J Sudo H. MicroRNA-8073: Tumor suppressor and potential therapeutic treatment. PLoS One (2018) 13(12):e0209750. 10.1371/journal.pone.0209750 30589909 PMC6307750

[95] Wang L Lu J Zhang H Lyu X Sun Z. MicroRNA-876-5p inhibits the progression of glioblastoma multiforme by directly targeting Forkhead box M1. Oncol Rep (2019) 41:702–10. 10.3892/or.2018.6804 30365126

[96] Dong G Pan T Zhou D Li C Liu J Zhang J. FBXL19-AS1 promotes cell proliferation and inhibits cell apoptosis via miR-876-5p/FOXM1 axis in breast cancer. Acta Biochim Biophys Sin (Shanghai) (2019) 51:1106–13. 10.1093/abbs/gmz110 31696201

[97] Zhou H Yang L Xu X Lu M Guo R Li D. miR-34a inhibits esophageal squamous cell carcinoma progression via regulation of FOXM1. Oncol Lett (2019) 17:706–12. 10.3892/ol.2018.9593 PMC6312991 30655820

[98] Cao X Liu L Cao X Cui Y Zou C Chen A. The DNMT1/miR-34a/FOXM1 Axis Contributes to Stemness of Liver Cancer Cells. J Oncol (2020) 2020:8978930. 10.1155/2020/8978930 32308683 PMC7142390

[99] Chen F Bai G Li Y Feng Y Wang L. A positive feedback loop of long noncoding RNA CCAT2 and FOXM1 promotes hepatocellular carcinoma growth. Am J Cancer Res (2017) 7:1423–34. PMC5523025 28744394

[100] Wei Y Sun Q Zhao L Wu J Chen X Wang Y. LncRNA UCA1-miR-507-FOXM1 axis is involved in cell proliferation, invasion and G0/G1 cell cycle arrest in melanoma. Med Oncol (2016) 33:88. 10.1007/s12032-016-0804-2 27389544

[101] Li X Chu H Lv T Wang L Kong S Dai S. miR-342-3p suppresses proliferation, migration and invasion by targeting FOXM1 in human cervical cancer. FEBS Lett (2014) 588:3298–307. 10.1016/j.febslet.2014.07.020 25066298

[102] Wang S-H Ma F Tang Z-H Wu X-C Cai Q Zhang M-D. Long non-coding RNA H19 regulates FOXM1 expression by competitively binding endogenous miR-342-3p in gallbladder cancer. J Exp Clin Cancer Res (2016) 35:160. 10.1186/s13046-016-0436-6 27716361 PMC5048611

[103] Zhao S Wang Y Lou Y Wang Y Sun J Luo M. MicroRNA−320a suppresses tumour cell proliferation and invasion of renal cancer cells by targeting FoxM1. Oncol Rep (2018) 40:1917–26. 10.3892/OR.2018.6597 PMC6111456 30066895

[104] Sun JY Zhao ZW Li WM Yang G Jing PY Li P. Knockdown of MALAT1 expression inhibits HUVEC proliferation by upregulation of miR-320a and downregulation of FOXM1 expression. Oncotarget (2017) 8:61499–509. 10.18632/oncotarget.18507 PMC5617440 28977880

[105] Luo X Wang GH Bian ZL Li XW Zhu BY Jin CJ. Long non-coding RNA CCAL/miR-149/FOXM1 axis promotes metastasis in gastric cancer. Cell Death Dis (2018) 9(10):993. 10.1038/s41419-018-0969-z 30250169 PMC6155366

[106] Ma J Qi G Li L. LncRNA NNT-AS1 promotes lung squamous cell carcinoma progression by regulating the miR-22/FOXM1 axis. Cell Mol Biol Lett (2020) 25:34. 10.1186/s11658-020-00227-8 32514270 PMC7257167

[107] Xiao J Lin L Luo D Shi L Chen W Fan H. Long noncoding RNA TRPM2-AS acts as a microRNA sponge of miR-612 to promote gastric cancer progression and radioresistance. Oncogenesis (2020) 9(3):29. 10.1038/s41389-020-0215-2 32123162 PMC7052141

[108] Gao F Feng J Yao H Li Y Xi J Yang J. LncRNA SBF2-AS1 promotes the progression of cervical cancer by regulating miR-361-5p/FOXM1 axis. Artif Cells Nanomed Biotechnol (2019) 47:776–82. 10.1080/21691401.2019.1577883 30856345

[109] Shen B Zhou N Hu T Zhao W Wu D Wang S. LncRNA MEG3 negatively modified osteosarcoma development through regulation of miR-361-5p and FoxM1. J Cell Physiol (2019) 234:13464–80. 10.1002/jcp.28026 PMC13483347 30624782

[110] Li CF Li YC Wang Y Sun LB. The Effect of LncRNA H19/miR-194-5p Axis on the Epithelial-Mesenchymal Transition of Colorectal Adenocarcinoma. Cell Physiol Biochem (2018) 50:214–32. 10.1159/000493968 30278464

[111] Yuan Y Haiying G Zhuo L Ying L Xin H. Long non-coding RNA LINC00339 facilitates the tumorigenesis of non-small cell lung cancer by sponging miR-145 through targeting FOXM1. BioMed Pharmacother (2018) 105:707–13. 10.1016/j.biopha.2018.06.022 29906749

[112] Wang G Wang X Jin Y. LINC01410/MIR-3619-5p/FOXM1 feedback loop regulates papillary thyroid carcinoma cell proliferation and apoptosis. Cancer Biother Radiopharm (2019) 34:572–80. 10.1089/cbr.2019.2854 31644316

[113] Wang W Guo P Chen M Chen D Cheng Y He L. FOXM1/LINC00152 feedback loop regulates proliferation and apoptosis in rheumatoid arthritis fibroblast-like synoviocytes via Wnt/β-catenin signaling pathway. Biosci Rep (2020) 40(1):BSR20191900. 10.1042/BSR20191900 31854447 PMC6974425

[114] Liu S Guo W Shi J Li N Yu X Xue J. MicroRNA-135a contributes to the development of portal vein tumor thrombus by promoting metastasis in hepatocellular carcinoma. J Hepatol (2012) 56:389–96. 10.1016/j.jhep.2011.08.008 21888875

[115] Li Y Zhang T Zhang Y Zhao X Wang W. Targeting the FOXM1-regulated long noncoding RNA TUG1 in osteosarcoma. Cancer Sci (2018) 109:3093–104. 10.1111/cas.13765 PMC6172046 30099814

[116] Xu M-D Wang Y Weng W Wei P Qi P Zhang Q. Biology of Human Tumors A Positive Feedback Loop of lncRNA-PVT1 and FOXM1 Facilitates Gastric Cancer Growth and Invasion. Clin Cancer Res (2017) 23(8):2071–80. 10.1158/1078-0432.CCR-16-0742 27756785

[117] Cohn O Feldman M Weil L Kublanovsky M Levy D. Chromatin associated SETD3 negatively regulates VEGF expression. Sci Rep (2016) 6:1–10. 10.1038/srep37115 27845446 PMC5109252

[118] Cao XJ Arnaudo AM Garcia BA. Large-scale global identification of protein lysine methylation in vivo. Epigenetics (2013) 8:477–85. 10.4161/epi.24547 PMC3741217 23644510

[119] Zhou Z Chen H Xie R Wang H Li S Xu Q. Epigenetically modulated FOXM1 suppresses dendritic cell maturation in pancreatic cancer and colon cancer. Mol Oncol (2019) 13:873–93. 10.1002/1878-0261.12443 PMC6441919 30628173

[120] Ma RYM. Raf/MEK/MAPK signaling stimulates the nuclear translocation and transactivating activity of FOXM1c. J Cell Sci (2005) 118:795–806. 10.1242/jcs.01657 15671063

[121] Westendorf JM Rao PN Gerace L. Cloning of cDNAs for M-phase phosphoproteins recognized by the MPM2 monoclonal antibody and determination of the phosphorylated epitope. Proc Natl Acad Sci U S A (1994) 91:714–8. 10.1073/pnas.91.2.714 PMC43019 8290587

[122] Major ML Lepe R Costa RH. Forkhead box M1B transcriptional activity requires binding of Cdk-cyclin complexes for phosphorylation-dependent recruitment of p300/CBP coactivators. Mol Cell Biol (2004) 24:2649–61. 10.1128/MCB.24.7.2649-2661.2004 PMC371108 15024056

[123] Laoukili J Alvarez M Meijer LAT Stahl M Mohammed S Kleij L. Activation of FoxM1 during G2 requires cyclin A/Cdk-dependent relief of autorepression by the FoxM1 N-terminal domain. Mol Cell Biol (2008) 28:3076–87. 10.1128/MCB.01710-07 PMC2293089 18285455

[124] Anders L Ke N Hydbring P Choi YJ Widlund HR Chick JM. A Systematic Screen for CDK4/6 Substrates Links FOXM1 Phosphorylation to Senescence Suppression in Cancer Cells. Cancer Cell (2011) 20:620–34. 10.1016/j.ccr.2011.10.001 PMC3237683 22094256

[125] Lüscher-Firzlaff JM Lilischkis R Lüscher B. Regulation of the transcription factor FOXM1c by Cyclin E/CDK2. FEBS Lett (2006) 580(7):1716–22. 10.1016/j.febslet.2006.02.021 16504183

[126] Chen Y-J Dominguez-Brauer C Wang Z Asara JM Costa RH Tyner AL. A Conserved Phosphorylation Site within the Forkhead Domain of FoxM1B Is Required for Its Activation by Cyclin-CDK1. J Biol Chem (2009) 284(44):30695–707. 10.1074/jbc.M109.007997 PMC2781623 19737929

[127] Mukhopadhyay NK Chand V Pandey A Kopanja D Carr JR Chen YJ. Plk1 Regulates the Repressor Function of FoxM1b by inhibiting its Interaction with the Retinoblastoma Protein. Sci Rep (2017) 7:46017. 10.1038/srep46017 28387346 PMC5384083

[128] Fu Z Malureanu L Huang J Wang W Li H Van Deursen JM. Plk1-dependent phosphorylation of FoxM1 regulates a transcriptional programme required for mitotic progression. Nat Cell Biol (2008) 10(9):1076–82. 10.1038/ncb1767 PMC2882053 19160488

[129] Zhang Z Zhang G Kong C. FOXM1 participates in PLK1-regulated cell cycle progression in renal cell cancer cells. Oncol Lett (2016) 11:2685–91. 10.3892/ol.2016.4228 PMC4812172 27073539

[130] Joshi K Banasavadi-Siddegowda Y Mo X Kim S-H Mao P Kig C. MELK-dependent FOXM1 phosphorylation is essential for proliferation of glioma stem cells. Stem Cells (2013) 31:1051–63. 10.1002/stem.1358 PMC3744761 23404835

[131] Wang R Song Y Xu X Wu Q Liu C. The expression of Nek7, FoxM1, and Plk1 in gallbladder cancer and their relationships to clinicopathologic features and survival. Clin Transl Oncol (2013) 15:626–32. 10.1007/s12094-012-0978-9 23359173

[132] Dibb M Han N Choudhury J Hayes S Valentine H West C. The FOXM1-PLK1 axis is commonly upregulated in oesophageal adenocarcinoma. Br J Cancer (2012) 107:1766–75. 10.1038/bjc.2012.424 PMC3493860 23037713

[133] Liu F Li N Liu Y Zhang J Zhang J Wang Z. Homeodomain interacting protein kinase-2 phosphorylates FOXM1 and promotes FOXM1-mediated tumor growth in renal cell carcinoma. J Cell Biochem (2019) 120:10391–401. 10.1002/jcb.28323 30609136

[134] Tan Y Raychaudhuri P Costa RH. Chk2 mediates stabilization of the FoxM1 transcription factor to stimulate expression of DNA repair genes. Mol Cell Biol (2007) 27:1007–16. 10.1128/MCB.01068-06 PMC1800696 17101782

[135] Wierstra I. The transcription factor FOXM1c is activated by protein kinase CK2, protein kinase A (PKA), c-Src and Raf-1. Biochem Biophys Res Commun (2011) 413:230–5. 10.1016/j.bbrc.2011.08.075 21875579

[136] Lv C Zhao G Sun X Wang P Xie N Luo J. Acetylation of FOXM1 is essential for its transactivation and tumor growth stimulation. Oncotarget (2016) 7:60366–82. 10.18632/oncotarget.11332 PMC5312389 27542221

[137] Hilgarth RS Murphy LA Skaggs HS Wilkerson DC Xing H Sarge KD. Regulation and function of SUMO modification. J Biol Chem (2004) 279:53899–902. 10.1074/jbc.R400021200 15448161

[138] Myatt SS Kongsema M Man CW-Y Kelly DJ Gomes AR Khongkow P. SUMOylation inhibits FOXM1 activity and delays mitotic transition. Oncogene (2014) 33:4316–29. 10.1038/onc.2013.546 PMC4096495 24362530

[139] Zhang J Yuan C Wu J Elsayed Z Fu Z. Polo-like kinase 1-mediated phosphorylation of Forkhead box protein M1b antagonizes its SUMOylation and facilitates its mitotic function. J Biol Chem (2015) 290:3708–19. 10.1074/jbc.M114.634386 PMC4319035 25533473

[140] Jaiswal N John R Chand V Nag A. Oncogenic Human Papillomavirus 16E7 modulates SUMOylation of FoxM1b. Int J Biochem Cell Biol (2015) 58:28–36. 10.1016/j.biocel.2014.11.002 25462159

[141] Wang C-M Liu R Wang L Nascimento L Brennan V Yang W-H. SUMOylation of FOXM1B Alters Its Transcriptional Activity on Regulation of MiR-200 Family and JNK1 in MCF7 Human Breast Cancer Cells. Int J Mol Sci (2014) 15:10233–51. 10.3390/ijms150610233 PMC4100150 24918286

[142] Schimmel J Eifler K Sigurðsson JO Cuijpers SAG Hendriks IA Verlaan-de Vries M. Uncovering SUMOylation Dynamics during Cell-Cycle Progression Reveals FoxM1 as a Key Mitotic SUMO Target Protein. Mol Cell (2014) 53:1053–66. 10.1016/j.molcel.2014.02.001 24582501

[143] Schrader EK Harstad KG Matouschek A. Targeting proteins for degradation. Nat Chem Biol (2009) 5(11):815–22. 10.1038/nchembio.250 PMC4228941 19841631

[144] Park HJ Costa RH Lau LF Tyner AL Raychaudhuri P. Anaphase-promoting complex/cyclosome-CDH1-mediated proteolysis of the forkhead box M1 transcription factor is critical for regulated entry into S phase. Mol Cell Biol (2008) 28:5162–71. 10.1128/MCB.00387-08 PMC2519738 18573889

[145] Jeffery J Kalimutho M Johansson P Cardenas D Kumar R Khanna K. FBXO31 protects against genomic instability by capping FOXM1 levels at the G2/M transition. Nat Publ Gr (2016) 36(7):1012–22. 10.1038/onc.2016.268 27568981

[146] Song Z Li J Zhang L Deng J Fang Z Xiang X. UCHL3 promotes pancreatic cancer progression and chemo-resistance through FOXM1 stabilization. Am J Cancer Res (2019) 9:1970–81. PMC6780670 31598398

[147] Li XY Wu HY Mao XF Jiang LX Wang YX. USP5 promotes tumorigenesis and progression of pancreatic cancer by stabilizing FoxM1 protein. Biochem Biophys Res Commun (2017) 492:48–54. 10.1016/j.bbrc.2017.08.040 28807830

[148] Arceci A Bonacci T Wang X Stewart K Damrauer JS Hoadley KA. FOXM1 Deubiquitination by USP21 Regulates Cell Cycle Progression and Paclitaxel Sensitivity in Basal-like Breast Cancer. Cell Rep (2019) 26:3076–86.e6. 10.1016/j.celrep.2019.02.054 30865895 PMC6425951

[149] Zhou K Mai H Zheng S Cai W Yang X Chen Z. OTUB1-mediated deubiquitination of FOXM1 up-regulates ECT-2 to promote tumor progression in renal cell carcinoma. Cell Biosci (2020) 10:50. 10.1186/s13578-020-00408-0 32257108 PMC7106863

[150] Kongsema M Zona S Karunarathna U Cabrera E Man EPS Yao S. RNF168 cooperates with RNF8 to mediate FOXM1 ubiquitination and degradation in breast cancer epirubicin treatment. Oncogenesis (2016) 5:e252. 10.1038/oncsis.2016.57 27526106 PMC5007831

[151] Chen Y Li Y Xue J Gong A Yu G Zhou A. Wnt induced deubiquitination FoxM1 ensures nucleus β catenin transactivation. EMBO J (2016) 35:668–84. 10.15252/embj.201592810 PMC4801947 26912724

[152] Wang X Krupczak-Hollis K Tan Y Dennewitz MB Adami GR Costa RH. Increased hepatic Forkhead Box M1B (FoxM1B) levels in old-aged mice stimulated liver regeneration through diminished p27Kip1 protein levels and increased Cdc25B expression. J Biol Chem (2002) 277:44310–6. 10.1074/jbc.M207510200 12221098

[153] Kalin TV Wang IC Ackerson TJ Major ML Detrisac CJ Kalinichenko VV. Increased levels of the FoxM1 transcription factor accelerate development and progression of prostate carcinomas in both TRAMP and LADY transgenic mice. Cancer Res (2006) 66:1712–20. 10.1158/0008-5472.CAN-05-3138 PMC1363687 16452231

[154] Wang I-C Chen Y-J Hughes DE Ackerson T Major ML Kalinichenko VV. FoxM1 Regulates Transcription of JNK1 to Promote the G 1 /S Transition and Tumor Cell Invasiveness. J Biol Chem (2008) 283(30):20770–8. 10.1074/jbc.M709892200 PMC2475715 18524773

[155] Petrovic V Costa RH Lau LF Raychaudhuri P Tyner AL. FoxM1 Regulates Growth Factor-induced Expression of Kinase-interacting Stathmin (KIS) to Promote Cell Cycle Progression. J Biol Chem (2008) 283:453–60. 10.1074/jbc.M705792200 17984092

[156] Sullivan C Liu Y Shen J Curtis A Newman C Hock JM. Novel Interactions between FOXM1 and CDC25A Regulate the Cell Cycle. PLoS One (2012) 7:e51277. 10.1371/journal.pone.0051277 23240008 PMC3519786

[157] Wang I-C Chen Y-J Hughes D Petrovic V Major ML Park HJ. Forkhead box M1 regulates the transcriptional network of genes essential for mitotic progression and genes encoding the SCF (Skp2-Cks1) ubiquitin ligase. Mol Cell Biol (2005) 25:10875–94. 10.1128/MCB.25.24.10875-10894.2005 PMC1316960 16314512

[158] Liu Y Gong Z Sun L Li X. FOXM1 and androgen receptor co-regulate CDC6 gene transcription and DNA replication in prostate cancer cells. Biochim Biophys Acta - Gene Regul Mech (2014) 1839:297–305. 10.1016/j.bbagrm.2014.02.016 24583551

[159] Park Y-Y Jung SY Jennings NB Rodriguez-Aguayo C Peng G Lee S-R. FOXM1 mediates Dox resistance in breast cancer by enhancing DNA repair. Carcinogenesis (2012) 33:1843–53. 10.1093/carcin/bgs167 PMC3529559 22581827

[160] Wang I-C Meliton L Ren X Zhang Y Balli D Snyder J. Deletion of Forkhead Box M1 Transcription Factor from Respiratory Epithelial Cells Inhibits Pulmonary Tumorigenesis. PLoS One (2009) 4:e6609. 10.1371/journal.pone.0006609 19672312 PMC2720537

[161] Leung TWC Lin SSW Tsang ACC Tong CSW Ching JCY Leung WY. Over-expression of FoxM1 stimulates cyclin B1 expression. FEBS Lett (2001) 507:59–66. 10.1016/S0014-5793(01)02915-5 11682060

[162] Krishnan A KD Babu PSS Jagadeeshan S Prasad M Nair SA. Oncogenic Actions of SKP2 Involves Deregulation of CDK1 Turnover Mediated by FOXM1. J Cell Biochem (2017) 118:797–807. 10.1002/jcb.25754 27684411

[163] Sanders DA Gormally MV Marsico G Beraldi D Tannahill D Balasubramanian S. FOXM1 binds directly to non-consensus sequences in the human genome. Genome Biol (2015) 16:130. 10.1186/s13059-015-0696-z 26100407 PMC4492089

[164] Bu H Li Y Jin C Yu H Wang X Chen J. Overexpression of PRC1 indicates a poor prognosis in ovarian cancer. Int J Oncol (2020) 56:685–96. 10.3892/ijo.2020.4959 PMC7010224 31922238

[165] Wang X Kiyokawa H Dennewitz MB Costa RH. The Forkhead Box m1b transcription factor is essential for hepatocyte DNA replication and mitosis during mouse liver regeneration. Proc Natl Acad Sci U S A (2002) 99:16881–6. 10.1073/pnas.252570299 PMC139238 12482952

[166] Tan Y Yoshida Y Hughes DE Costa RH. Increased Expression of Hepatocyte Nuclear Factor 6 Stimulates Hepatocyte Proliferation During Mouse Liver Regeneration. Gastroenterology (2006) 130:1283–300. 10.1053/j.gastro.2006.01.010 PMC1440887 16618419

[167] Sengupta A Kalinichenko VV Yutzey KE. FoxO1 and FoxM1 transcription factors have antagonistic functions in neonatal cardiomyocyte cell-cycle withdrawal and IGF1 gene regulation. Circ Res (2013) 112:267–77. 10.1161/CIRCRESAHA.112.277442 PMC3548965 23152492

[168] Dai B Pieper RO Li D Wei P Liu M Woo SY. FoxM1B regulates NEDD4-1 expression, leading to cellular transformation and full malignant phenotype in immortalized human astrocytes. Cancer Res (2010) 70:2951–61. 10.1158/0008-5472.CAN-09-3909 PMC2848915 20332230

[169] Monteiro LJ Khongkow P Kongsema M Morris JR Man C Weekes D. The Forkhead Box M1 protein regulates BRIP1 expression and DNA damage repair in epirubicin treatment. Oncogene (2013) 32:4634–45. 10.1038/onc.2012.491 PMC3874579 23108394

[170] Zona S Bella L Burton MJ Nestal de Moraes G Lam EW-F. FOXM1: an emerging master regulator of DNA damage response and genotoxic agent resistance. Biochim Biophys Acta (2014) 1839:1316–22. 10.1016/j.bbagrm.2014.09.016 PMC4316173 25287128

[171] Laoukili J Kooistra MRH Brás A Kauw J Kerkhoven RM Morrison A. FoxM1 is required for execution of the mitotic programme and chromosome stability. Nat Cell Biol (2005) 7:126–36. 10.1038/ncb1217 15654331

[172] Puig-Butille JA Vinyals A Ferreres JR Aguilera P Cabré E Tell-Martí G. AURKA Overexpression Is Driven by FOXM1 and MAPK/ERK Activation in Melanoma Cells Harboring BRAF or NRAS Mutations: Impact on Melanoma Prognosis and Therapy. J Invest Dermatol (2017) 137:1297–310. 10.1016/j.jid.2017.01.021 28188776

[173] Bonet C Giuliano S Ohanna M Bille K Allegra M Lacour JP. Aurora B is regulated by the mitogen-activated protein kinase/extracellular signal-regulated kinase (MAPK/ERK) signaling pathway and is a valuable potential target in melanoma cells. J Biol Chem (2012) 287:29887–98. 10.1074/jbc.M112.371682 PMC3436153 22767597

[174] Hu G Yan Z Zhang C Cheng M Yan Y Wang Y. FOXM1 promotes hepatocellular carcinoma progression by regulating KIF4A expression. J Exp Clin Cancer Res (2019) 38:188. 10.1186/s13046-019-1202-3 31072351 PMC6507024

[175] Thiru P Kern DM McKinley KL Monda JK Rago F Su K-C. Kinetochore genes are coordinately up-regulated in human tumors as part of a FoxM1-related cell division program. Mol Biol Cell (2014) 25:1983–94. 10.1091/mbc.E14-03-0837 PMC4072572 24829384

[176] Halasi M Gartel AL. Suppression of FOXM1 sensitizes human cancer cells to cell death induced by DNA-damage. PLoS One (2012) 7(2):e31761. 10.1371/journal.pone.0031761 22393369 PMC3290538

[177] Nestal de Moraes G Delbue D Silva KL Robaina MC Khongkow P Gomes AR. FOXM1 targets XIAP and Survivin to modulate breast cancer survival and chemoresistance. Cell Signal (2015) 27:2496–505. 10.1016/j.cellsig.2015.09.013 26404623

[178] Hamurcu Z Delibaşı N Nalbantoglu U Sener EF Nurdinov N Tascı B. FOXM1 plays a role in autophagy by transcriptionally regulating Beclin-1 and LC3 genes in human triple-negative breast cancer cells. J Mol Med (2019) 97:491–508. 10.1007/s00109-019-01750-8 30729279

[179] Park HJ Carr JR Wang Z Nogueira V Hay N Tyner AL. FoxM1, a critical regulator of oxidative stress during oncogenesis. EMBO J (2009) 28:2908–18. 10.1038/emboj.2009.239 PMC2760115 19696738

[180] Cui J Shi M Xie D Wei D Jia Z Zheng S. FOXM1 Promotes the Warburg Effect and Pancreatic Cancer Progression via Transactivation of LDHA Expression. Clin Cancer Res (2014) 20:2595–606. 10.1158/1078-0432.CCR-13-2407 PMC4024335 24634381

[181] Bala Bhaskara Rao K Katragunta K Sarma UM Jain N. Abundance of d-2-hydroxyglutarate in G2/M is determined by FOXM1 in mutant IDH1-expressing cells. FEBS Lett (2019) 593:2177–93. 10.1002/1873-3468.13500 31211872

[182] Wang Y Yun Y Wu B Wen L Wen M Yang H. FOXM1 promotes reprogramming of glucose metabolism in epithelial ovarian cancer cells via activation of GLUT1 and HK2 transcription. Oncotarget (2016) 7:47985–97. 10.18632/oncotarget.10103 PMC5216994 27351131

[183] Black M Arumugam P Shukla S Pradhan A Ustiyan V Milewski D. FOXM1 nuclear transcription factor translocates into mitochondria and inhibits oxidative phosphorylation. Mol Biol Cell (2020) 31:1411–24. 10.1091/MBC.E19-07-0413 PMC7353143 32348194

[184] Zhang Y Zhang N Dai B Liu M Sawaya R Xie K. FoxM1B transcriptionally regulates vascular endothelial growth factor expression and promotes the angiogenesis and growth of glioma cells. Cancer Res (2008) 68:8733–42. 10.1158/0008-5472.CAN-08-1968 PMC2597644 18974115

[185] Luo X Yao J Nie P Yang Z Feng H Chen P. FOXM1 promotes invasion and migration of colorectal cancer cells partially dependent on HSPA5 transactivation. Oncotarget (2016) 7:26480–95. 10.18632/oncotarget.8419 PMC5041994 27034162

[186] Huang C Xie D Cui J Li Q Gao Y Xie K. FOXM1c promotes pancreatic cancer epithelial-to-mesenchymal transition and metastasis via upregulation of expression of the urokinase plasminogen activator system. Clin Cancer Res (2014) 20:1477–88. 10.1158/1078-0432.CCR-13-2311 PMC3959252 24452790

[187] Raychaudhuri P Park HJ. FoxM1: A master regulator of tumor metastasis. Cancer Res (2011) 71:4329–33. 10.1158/0008-5472.CAN-11-0640 PMC3129416 21712406

[188] Balli D Ustiyan V Zhang Y Wang IC Masino AJ Ren X. Foxm1 transcription factor is required for lung fibrosis and epithelial-to-mesenchymal transition. EMBO J (2013) 32:231–44. 10.1038/emboj.2012.336 PMC3553386 23288041

[189] Hamurcu Z Ashour A Kahraman N Ozpolat B. FOXM1 regulates expression of eukaryotic elongation factor 2 kinase and promotes proliferation, invasion and tumorgenesis of human triple negative breast cancer cells. Oncotarget (2016) 7:16619–35. 10.18632/oncotarget.7672 PMC4941339 26918606

[190] Wierstra I. The transcription factor FOXM1c binds to and transactivates the promoter of the tumor suppressor gene E-cadherin. Cell Cycle (2011) 10:760–6. 10.4161/cc.10.5.14827 21311221

[191] Lee Y Kim KH Kim DG Cho HJ Kim Y Rheey J. FoxM1 Promotes Stemness and Radio-Resistance of Glioblastoma by Regulating the Master Stem Cell Regulator Sox2. PLoS One (2015) 10:e0137703. 10.1371/journal.pone.0137703 26444992 PMC4596841

[192] Wang K Zhu X Yin Y. Maslinic Acid Enhances Docetaxel Response in Human Docetaxel-Resistant Triple Negative Breast Carcinoma MDA-MB-231 Cells via Regulating MELK-FoxM1-ABCB1 Signaling Cascade. Front Pharmacol (2020) 11:835. 10.3389/fphar.2020.00835 32581798 PMC7295941

[193] Zhu X Xue L Yao Y Wang K Tan C Zhuang M. The FoxM1-ABCC4 axis mediates carboplatin resistance in human retinoblastoma Y-79 cells. Acta Biochim Biophys Sin (Shanghai) (2018) 50:914–20. 10.1093/abbs/gmy080 30060118

[194] Hou Y Zhu Q Li Z Peng Y Yu X Yuan B. The FOXM1–ABCC5 axis contributes to paclitaxel resistance in nasopharyngeal carcinoma cells. Cell Death Dis (2017) 8:e2659. 10.1038/cddis.2017.53 28277541 PMC5386553

[195] Khongkow P Karunarathna U Khongkow M Gong C Gomes AR Yagüe E. FOXM1 targets NBS1 to regulate DNA damage-induced senescence and epirubicin resistance. Oncogene (2014) 33:4144–55. 10.1038/onc.2013.457 PMC3969838 24141789

[196] Kruiswijk F Hasenfuss SC Sivapatham R Baar MP Putavet D Naipal KAT. Targeted inhibition of metastatic melanoma through interference with Pin1-FOXM1 signaling. Oncogene (2016) 35:2166–77. 10.1038/onc.2015.282 PMC4757516 26279295

[197] Maachani UB Shankavaram U Kramp T Tofilon PJ Camphausen K Tandle AT. FOXM1 and STAT3 interaction confers radioresistance in glioblastoma cells. Oncotarget (2016) 7:77365–77. 10.18632/oncotarget.12670 PMC5340228 27764801

[198] Xue J Lin X Chiu W-T Chen Y-H Yu G Liu M. Sustained activation of SMAD3/SMAD4 by FOXM1 promotes TGF-β–dependent cancer metastasis. J Clin Invest (2014) 124:564–79. 10.1172/JCI71104 PMC3904622 24382352

[199] Bhat UG Jagadeeswaran R Halasi M Gartel AL. Nucleophosmin Interacts with FOXM1 and Modulates the Level and Localization of FOXM1 in Human Cancer Cells. J Biol Chem (2011) 48:41425 –33. 10.1074/jbc.M111.270843 PMC3308854 21979956

[200] Zhang N Wei P Gong A Chiu WT Te LH Colman H. FoxM1 Promotes β-Catenin Nuclear Localization and Controls Wnt Target-Gene Expression and Glioma Tumorigenesis. Cancer Cell (2011) 20:427–42. 10.1016/j.ccr.2011.08.016 PMC3199318 22014570

[201] Liu J Guo S Li Q Yang L Xia Z Zhang L. Phosphoglycerate dehydrogenase induces glioma cells proliferation and invasion by stabilizing forkhead box M1. J Neurooncol (2013) 111:245–55. 10.1007/s11060-012-1018-x PMC3565087 23229761

[202] Halasi M Váraljai R Benevolenskaya E Gartel AL. A novel function of molecular chaperone HSP70: Suppression of oncogenic FOXM1 after proteotoxic stress. J Biol Chem (2016) 291:142–8. 10.1074/jbc.M115.678227 PMC4697151 26559972

[203] Hwang S Lee H-J Jung J Sim D Hwang J Park J. Inhibition of Wnt3a/FOXM1/β-Catenin Axis and Activation of GSK3β and Caspases are Critically Involved in Apoptotic Effect of Moracin D in Breast Cancers. Int J Mol Sci (2018) 19:2681. 10.3390/ijms19092681 PMC6164368 30201862

[204] Yang L He K Yan S Yang Y Gao X Zhang M. Metadherin/astrocyte elevated gene-1 positively regulates the stability and function of forkhead box M1 during tumorigenesis. Neuro Oncol (2017) 19:352–63. 10.1093/neuonc/now229 PMC5464332 27923917

[205] Khan I Zia M Halasi M Gann P Gaitonde S Gartel A. FOXM1 Binds Nucleophosmin in AML and Confers Resistance to Chemotherapy. Blood (2015) 126:2467–7. 10.1182/blood.v126.23.2467.2467

[206] Wang Y Zhou X Xu M Weng W Zhang Q Yang Y. OTUB1-catalyzed deubiquitination of FOXM1 facilitates tumor progression and predicts a poor prognosis in ovarian cancer. Oncotarget (2016) 7:36681–97. 10.18632/oncotarget.9160 PMC5095031 27167337

[207] Karunarathna U Kongsema M Zona S Gong C Cabrera E Gomes A. OTUB1 inhibits the ubiquitination and degradation of FOXM1 in breast cancer and epirubicin resistance. Oncogene (2015) 35:1433–44. 10.1038/onc.2015.208 PMC4606987 26148240

[208] Kalinichenko VV Major ML Wang X Petrovic V Kuechle J Yoder HM. Foxm1b transcription factor is essential for development of hepatocellular carcinomas and is negatively regulated by the p19ARF tumor suppressor. Genes Dev (2004) 18:830–50. 10.1101/gad.1200704 PMC387422 15082532

[209] Wierstra I Alves J. Transcription factor FOXM1c is repressed by RB and activated by cyclin D1/Cdk4. Biol Chem (2006) 387:949–62. 10.1515/BC.2006.119 16913845

[210] Carr JR Kiefer MM Park HJ Li J Wang Z Fontanarosa J. FoxM1 Regulates Mammary Luminal Cell Fate. Cell Rep (2012) 1:715–29. 10.1016/j.celrep.2012.05.005 PMC3401379 22813746

[211] Xu K Shu H-KG. Transcription factor interactions mediate EGF-dependent COX-2 expression. Mol Cancer Res (2013) 11:875–86. 10.1158/1541-7786.MCR-12-0706 PMC3748214 23635401

[212] Petrovic V Costa RH Lau LF Raychaudhuri P Tyner AL. Negative regulation of the oncogenic transcription factor FoxM1 by thiazolidinediones and mithramycin. Cancer Biol Ther (2010) 9:1008–16. 10.4161/cbt.9.12.11710 PMC3005150 20372080

[213] Dong GZ Jeong JH Lee YI Lee SY Zhao HY Jeon R. Diarylheptanoids suppress proliferation of pancreatic cancer PANC-1 cells through modulating shh-Gli-FoxM1 pathway. Arch Pharm Res (2017) 40:509–17. 10.1007/s12272-017-0905-2 28258481

[214] Halasi M Hitchinson B Shah BN Váraljai R Khan I Benevolenskaya EV. Honokiol is a FOXM1 antagonist article. Cell Death Dis (2018) 9:1–8. 10.1038/s41419-017-0156-7 29367668 PMC5833612

[215] Jiang L Cao XC Cao JG Liu F Quan MF Sheng XF. Casticin induces ovarian cancer cell apoptosis by repressing FoxM1 through the activation of FOXO3a. Oncol Lett (2013) 5:1605–10. 10.3892/ol.2013.1258 PMC3678892 23761826

[216] Bi Z Liu W Ding R Wu Y Dou R Zhang W. A novel peptide, 9R-P201, strongly inhibits the viability, proliferation and migration of liver cancer HepG2 cells and induces apoptosis by down-regulation of FoxM1 expression. Eur J Pharmacol (2017) 796:175–89. 10.1016/j.ejphar.2016.12.029 28012972

[217] Xiang Q Tan G Jiang X Wu K Tan W Tan Y. Suppression of FOXM1 Transcriptional Activities via a Single-Stranded DNA Aptamer Generated by SELEX. Sci Rep (2017) 7:45377. 10.1038/srep45377 28358012 PMC5371818

[218] Bhat UG Halasi M Gartel AL. FoxM1 Is a General Target for Proteasome Inhibitors. PLoS One (2009) 4:e6593. 10.1371/journal.pone.0006593 19672316 PMC2721658

[219] Pandit B Gartel AL. Cell Cycle FoxM1 knockdown sensitizes human cancer cells to proteasome inhibitor-induced apoptosis but not to autophagy. Cell Cycle (2011) 10(19):3269–73. 10.4161/cc.10.19.17735 PMC3233624 21941087

[220] Bhat UG Halasi M Gartel AL. Thiazole antibiotics target FoxM1 and induce apoptosis in human cancer cells. PLoS One (2009) 4:5592. 10.1371/journal.pone.0005592 PMC2680058 19440351

[221] Liu Y Zhu L Wen T Wan J Lei Y Chen H. [Forkhead domain inhibitor-6 (FDI-6) increases apoptosis and inhibits invasion and migration of laryngeal carcinoma cells by down-regulating nuclear FoxM1]. Xi Bao Yu Fen Zi Mian Yi Xue Za Zhi (2017) 33:611–6. 28502298

[222] Shukla S Milewski D Pradhan A Rama N Rice K Le T. The FOXM1 inhibitor RCM-1 decreases carcinogenesis and nuclear b-catenin. Mol Cancer Ther (2019) 18:1217–29. 10.1158/1535-7163.MCT-18-0709 PMC7341442 31040162

[223] Liu LP Cao XC Liu F Quan MF Sheng XF Ren KQ. Casticin induces breast cancer cell apoptosis by inhibiting the expression of forkhead box protein M1. Oncol Lett (2014) 7:1711–7. 10.3892/ol.2014.1911 PMC3997681 24765206

[224] He L Yang X Cao X Liu F Quan M Cao J. Casticin induces growth suppression and cell cycle arrest through activation of FOXO3a in hepatocellular carcinoma. Oncol Rep (2013) 29:103–8. 10.3892/or.2012.2076 23064420

[225] Barsotti AM Prives C. Pro-proliferative FoxM1 is a target of p53-mediated repression. Oncogene (2009) 28:4295–305. 10.1038/onc.2009.282 PMC2898139 19749794

[226] Ning Y Li Q Xiang H Liu F Cao J. Apoptosis induced by 7-difluoromethoxyl-5,4′-di-n-octyl genistein via the inactivation of FoxM1 in ovarian cancer cells. Oncol Rep (2012) 27:1857–64. 10.3892/or.2012.1739 22447287

[227] Xiang HL Liu F Quan MF Cao JG Lv Y. 7-difluoromethoxyl-5,4’-di-n-octylgenistein inhibits growth of gastric cancer cells through downregulating forkhead box M1. World J Gastroenterol (2012) 18:4618–26. 10.3748/wjg.v18.i33.4618 PMC3435790 22969238

[228] Ahmad A Ali S Wang Z Ali AS Sethi S Sakr WA. 3,3′-diindolylmethane enhances taxotere-induced growth inhibition of breast cancer cells through downregulation of FoxM1. Int J Cancer (2011) 129:1781–91. 10.1002/ijc.25839 PMC3803115 21154750

[229] Jin H Li XJ Park MH Kim SM. FOXM1-mediated downregulation of uPA and MMP9 by 3,3’-diindolylmethane inhibits migration and invasion of human colorectal cancer cells. Oncol Rep (2015) 33:3171–7. 10.3892/or.2015.3938 25962429

[230] Jin H Park MH Kim SM. 3,3′-Diindolylmethane potentiates paclitaxel-induced antitumor effects on gastric cancer cells through the Akt/FOXM1 signaling cascade. Oncol Rep (2015) 33:2031–6. 10.3892/or.2015.3758 25633416

[231] Gormally MV Dexheimer TS Marsico G Sanders DA Lowe C Matak-Vincović D. Suppression of the FOXM1 transcriptional program via novel small molecule inhibition. Nat Commun (2014) 5:5165. 10.1038/ncomms6165 25387393 PMC4258842

[232] Wang Z Ahmad A Banerjee S Azmi A Kong D Li Y. FoxM1 is a novel target of a natural agent in pancreatic cancer. Pharm Res (2010) 27:1159–68. 10.1007/s11095-010-0106-x PMC2975383 20354770

[233] Fu Z Cao X Liu L Cao X Cui Y Li X. Genistein inhibits lung cancer cell stem.like characteristics by modulating MnSOD and FoxM1 expression. Oncol Lett (2020) 20:2506–15. 10.3892/ol.2020.11802 PMC7400602 32782570

[234] Ketola K Munuganti RSN Davies A Nip KM Bishop JL Zoubeidi A. Targeting prostate cancer subtype 1 by Forkhead box M1 pathway inhibition. Clin Cancer Res (2017) 23:6923–33. 10.1158/1078-0432.CCR-17-0901 28899970

[235] Li Y Ligr M McCarron JP Daniels G Zhang D Zhao X. Natura-alpha targets forkhead box M1 and inhibits androgen-dependent and -independent prostate cancer growth and invasion. Clin Cancer Res (2011) 17:4414–24. 10.1158/1078-0432.CCR-11-0431 PMC3196615 21606178

[236] Arora R Yates C Gary BD McClellan S Tan M Xi Y. Panepoxydone targets NF-kB and FOXM1 to inhibit proliferation, induce apoptosis and reverse epithelial to mesenchymal transition in breast cancer. PLoS One (2014) 9:e98370. 10.1371/journal.pone.0098370 24896091 PMC4045585

[237] Bhat UG Zipfel PA Tyler DS Gartel AL. Novel anticancer compounds induce apoptosis in melanoma cells. Cell Cycle (2008) 7:1851–5. 10.4161/cc.7.12.6032 18583930

[238] Pratheeshkumar P Divya SP Parvathareddy SK Alhoshani NM Al-Badawi IA Tulbah A. FoxM1 and β-catenin predicts aggressiveness in Middle Eastern ovarian cancer and their co-targeting impairs the growth of ovarian cancer cells. Oncotarget (2018) 9:3590–604. 10.18632/oncotarget.23338 PMC5790485 29423068

[239] Ju SY Huang CYF Huang WC Su Y. Identification of thiostrepton as a novel therapeutic agent that targets human colon cancer stem cells. Cell Death Dis (2015) 6(7):e1801. 10.1038/cddis.2015.155 26136074 PMC4650716

[240] Tabatabaei Dakhili SA Pérez DJ Gopal K Haque M Ussher JR Kashfi K. SP1-independent inhibition of FOXM1 by modified thiazolidinediones. Eur J Med Chem (2020) 209:112902. 10.1016/j.ejmech.2020.112902 33069434

[241] Nakamura S Yamashita M Yokota D Hirano I Ono T Fujie M. Development and pharmacologic characterization of deoxybromophospha sugar derivatives with antileukemic activity. Invest New Drugs (2010) 28:381–91. 10.1007/s10637-009-9255-3 19436953

[242] Chan DW Hui WWY Cai PCH Liu MX Yung MMH Mak CSL. Targeting GRB7/ERK/FOXM1 Signaling Pathway Impairs Aggressiveness of Ovarian Cancer Cells. PLoS One (2012) 7:e52578. 10.1371/journal.pone.0052578 23285101 PMC3527599

[243] Madureira PA Varshochi R Constantinidou D Francis RE Coombes RC Yao KM. The forkhead box M1 protein regulates the transcription of the estrogen receptor α in breast cancer cells. J Biol Chem (2006) 281:25167–76. 10.1074/jbc.M603906200 16809346

[244] Wang JS Ren TN Xi T. Ursolic acid induces apoptosis by suppressing the expression of FoxM1 in MCF-7 human breast cancer cells. Med Oncol (2012) 29:10–5. 10.1007/s12032-010-9777-8 21191671

[245] Lok GTM Chan DW Liu VWS Hui WWY Leung THY Yao KM. Aberrant Activation of ERK/FOXM1 Signaling Cascade Triggers the Cell Migration/Invasion in Ovarian Cancer Cells. PLoS One (2011) 6:e23790. 10.1371/journal.pone.0023790 21858223 PMC3157468

[246] Carr JR Park HJ Wang Z Kiefer MM Raychaudhuri P. FoxM1 mediates resistance to herceptin and paclitaxel. Cancer Res (2010) 70:5054–63. 10.1158/0008-5472.CAN-10-0545 PMC2893542 20530690

[247] Li X Yao R Yue L Qiu W Qi W Liu S. FOXM1 mediates resistance to docetaxel in gastric cancer via up-regulating Stathmin. J Cell Mol Med (2014) 18:811–23. 10.1111/jcmm.12216 PMC4119387 24628949

